# Surface-based image reconstruction optimization for high-density functional near-infrared spectroscopy

**DOI:** 10.1117/1.NPh.13.2.025001

**Published:** 2026-03-14

**Authors:** Laura B. Carlton, Miray Altınkaynak, Shannon M. Kelley, Bernhard B. Zimmermann, Sreekanth Kura, Eike Middell, Alexander von Lühmann, Emily P. Stephen, Meryem A. Yücel, David A. Boas

**Affiliations:** a Boston University, Neurophotonics Center, Department of Biomedical Engineering, Boston, Massachusetts, United States; b Technische Universität Berlin, Intelligent Biomedical Sensing Lab, Berlin, Germany; c Berlin Institute for the Foundations of Learning and Data, Berlin, Germany; d Boston University, Department of Mathematics and Statistics, Boston, Massachusetts, United States

**Keywords:** functional near-infrared spectroscopy, diffuse optical tomography, human brain function, inverse problem

## Abstract

**Significance:**

Diffuse optical tomography (DOT) enables mapping of functional near-infrared spectroscopy channel-based optical density changes to spatial images of oxy- and deoxyhemoglobin. Accurate reconstruction requires optimization for specific probe geometries. Although prior work focused on volumetric voxel reconstructions with grid arrays, here we examine high-density hexagonal arrays for surface-based reconstructions of the brain and scalp.

**Aim:**

We evaluate measurement and spatial regularization, spatial basis functions, and reconstruction strategies to reduce crosstalk and improve localization. Both single-wavelength (indirect) and dual-wavelength (direct) approaches are compared.

**Approach:**

Simulations with a white-noise model guided parameter optimization using image quality metrics. Resting-state data were augmented with synthetic hemodynamic response functions (HRFs) to incorporate real measurement variance into the parameter optimization pipeline, and results were validated with a ball-squeezing motor task.

**Results:**

Gaussian spatial bases reduced brain–scalp crosstalk but lowered contrast-to-noise ratio and increased localization error. Indirect hemoglobin reconstruction decreased oxy–deoxy crosstalk. Validation data showed strong, lateralized motor cortex activation contralateral to the active hand.

**Conclusions:**

High-density hexagonal arrays enable accurate surface DOT reconstructions when optimized. Resting-state data augmented with synthetic HRFs provide an effective strategy for parameter selection, yielding localized activation with a high contrast-to-noise ratio.

## 1 Introduction

Neuroimaging has long been an essential tool for advancing our understanding of temporal and spatial activity in the brain during different tasks, cognitive states, and neurological conditions. Since its development, functional near-infrared spectroscopy (fNIRS) has emerged as an important neuroimaging modality as it enables noninvasive monitoring of changes in oxy- and deoxyhemoglobin concentration on the cortical surface of the brain.^1^ Like fMRI, fNIRS relies on the phenomenon of neurovascular coupling, wherein neuronal activation increases the local energy demand, which in turn induces a rise in regional cerebral blood flow. This will lead to a disproportionate increase in blood flow relative to oxygen consumption, resulting in a net increase in oxyhemoglobin levels.^2^ Oxy- and deoxyhemoglobin (HbO and HbR) exhibit distinct light absorption characteristics in the near-infrared spectrum. fNIRS leverages these differences to measure concentration changes in HbO and HbR and infer brain activity.^1^^,^^3^

Recent technological strides have led to the development of lightweight, wearable fNIRS systems, facilitating studies where participants can move freely and engage in more complex tasks.^4^^–^^7^ Although fNIRS has poor spatial resolution due to the limited depth penetration of near-infrared light, it offers relatively high temporal resolution and is robust against motion artefacts. These characteristics present distinct advantages over traditional neuroimaging techniques, particularly in novel, real-world experiments.^8^

Data collection is performed in channel space, where a channel is defined as a measurement utilizing a given source-detector pair for a given wavelength. The source emits near-infrared light at a specific wavelength, and the intensity of light after propagating through the head tissue is recorded at the detector. Using two wavelengths allows these measurements of light intensity to then be translated to changes in HbO and HbR using the modified Beer–Lambert law.^9^^,^^10^

Each fNIRS probe is composed of a network of channels. Traditional sparse probes are comprised of non-overlapping source-detector pairs with separations of ∼30  mm. Although sparse fNIRS probes can provide a coarse spatial sampling of cortical hemodynamic changes, advances in hardware have made it possible to build high-density (HD) systems that have overlapping channels with varying source-detector separations. This increases the spatial sampling density and improves the spatial sensitivity of the fNIRS measurements. Coinciding with the development of diffuse optical tomography (DOT), it is now possible to create 3D spatial maps of hemoglobin concentration changes, helping to distinguish changes in the brain from changes in the overlying scalp.^1^^,^^11^^–^^13^ Many researchers have leveraged this to investigate different cognitive responses, including visual stimulus,^14^^,^^15^ cognitive load,^16^^,^^17^ sensory and motor stimuli,^18^^,^^19^ and functional networks.^20^^–^^22^

Several systems have been designed to achieve this high-density design, which improves the spatial sampling of the device.^23^^–^^26^ In this paper, we utilize a whole-head (WH) high-density (HD) hexagonal optode array.^27^ The hexagonal arrangement of optodes improves the duty cycle of the temporal source multiplexing strategy used during data collection while maintaining HD optode packing. Furthermore, varying source-detector separations are achieved as each source has its first nearest-neighbor channels at 19 mm and second nearest-neighbor channels at 33 mm. The whole-head coverage provides the unique opportunity to study hemodynamic fluctuations across the entire cortical surface simultaneously. The process of DOT has been previously optimized for HD grid arrays,^14^^,^^28^^,^^29^ but the optimization of image quality for an HD hexagonal array has not yet been evaluated.

DOT further benefits from the use of anatomical head models to distinguish the various tissue types encountered by photons travelling between a source–detector pair. Recorded data contain information related to the hemodynamic changes within the brain associated with neural activity as well as systemic extra-cerebral hemoglobin changes occurring in the scalp. Historically, DOT has performed the image reconstruction with voxels spanning the layers of the head to achieve varying depth resolutions and isolate activity localized to the cortex.^12^ It has been shown that DOT can instead be performed using 3D surface representations of the brain and the scalp.^30^ Reconstructing images on surface vertices rather than within voxels is a flexible approach to introducing spatial priors. The method reconstructs images on the surface of the brain and head by first assigning each voxel to the nearest surface vertex. It is assumed that the absorption change is uniform within the voxels linked to the same vertex. This recognizes that the depth resolution of DOT challenges its ability to resolve hemoglobin changes occurring within individual tissue layers while also substantially reducing the number of free parameters in the image reconstruction.

DOT works on the principles of using a forward model to map changes in absorption on the vertices of the cortical and scalp surfaces to measured changes in optical density. The changes in optical density are given by the fNIRS measurements. To obtain the vertex-level absorption changes, one needs to solve the inverse problem. This can be done by reconstructing the optical density measurements for one wavelength at a time, called the indirect method, or both wavelengths simultaneously, called the direct method. The effect of these two approaches was evaluated in Ref. 31 for a transmission geometry. Here, we compare their effect on image quality for a reflection geometry. In addition, to reduce the dimensionality of the reconstruction problem, spatial basis functions can be implemented easily on the surface vertices by using 2D Gaussian kernels.^32^ These types of priors can also be implemented with a voxel-based image reconstruction, as has been partially done in Ref. 19, but the full approach we utilize here is more conveniently implemented with surfaces.

The quality of the images obtained through DOT will impact the ability of fNIRS to provide insights into neuroscientific questions and neurological disorders. This motivates the need for a comprehensive method to optimize the DOT pipeline. In this work, the DOT pipeline is optimized for the WH, HD hexagonal fNIRS probe using image reconstructions of the brain and scalp surfaces. The optimization begins with a simulation study—first using purely simulated data and then with resting-state data augmented with a synthetic hemodynamic response function (HRF)—and is finally validated using ball-squeezing motor task data. We found that using spatial basis functions improved the spatial crosstalk between the brain and scalp surfaces if the standard deviation of the Gaussian kernels used on the scalp was greater than 1 mm. This came at the expense of the contrast-to-noise ratio, which was improved when spatial basis functions were not used. We also observed a reduction in the chromophore crosstalk between HbO and HbR while using the indirect method versus the direct method for image reconstruction at the expense of a small reduction in contrast to noise ratio. These findings during the simulation study were consistent with the results from the ball-squeezing experiment. This thorough investigation of DOT for an HD hexagonal probe will provide a framework for the optimization of the DOT pipeline that can be extended to any system or probe geometry. Here, we further implemented a group averaging approach that circumvents the need for channel pruning by performing variance weighting. This has been described previously as best practice for obtaining reliable group results and statistics^33^^,^^34^ but has not been commonly implemented in fNIRS research.

## 2 Methods

### 2.1 Diffuse Optical Tomography Theory

DOT allows for the optical density changes in channel space to be mapped into absorption changes, $\delta \mu_{a}$, throughout the head. To do this, a sensitivity matrix, A, is generated for each wavelength using a Monte Carlo simulation, which models the probabilistic path of photons emitted from each optode through tissue in a 3D head model to get the fluence.^35^^–^^37^ From there, the sensitivity of a measurement channel to changes in absorption throughout the head is obtained by multiplying the fluence for a source by that from a detector forming that given channel. The volumetric fluence profile in the cortical grey matter is then projected onto the pial surface. Each voxel in the head model is assigned to the closest surface vertex. The sensitivity at a given vertex is the sum of all the voxels assigned. This requires the assumption that absorption changes in grey matter closest to the pial vertex are uniform.^30^ A similar projection can be performed from voxels in the extracerebral tissue to the scalp surface.

Each row in the sensitivity matrix A represents the sensitivity profile of a given channel, so the dimensions of the final matrix are the number of measurement channels ($n_{\text{channels}}$) by number of vertices ($n_{\text{vertices}}$) in the head model.^36^^,^^37^ This results in the following forward model for a given wavelength $\lambda_{i}$:

$$y_{\Delta \mathrm{OD}}(\lambda_{i})=A(\lambda_{i})x_{\delta \mu_{a}}(\lambda_{i}),$$ (1)

where $y_{\Delta \mathrm{OD}}(\lambda_{i})$ is a vector of channel measurements ($n_{\text{channels}}$) at wavelength $\lambda_{i}$ and $x_{\delta \mu_{a}}(\lambda_{i})$ is a vector of the absorption changes on the vertices within the head ($n_{\text{vertices}}$). For more details of how this sensitivity matrix is obtained on the surface vertices, please see Ref. 30.

The inverse problem can be used to solve for $x_{\delta \mu_{a}}(\lambda_{i})$ for each wavelength independently:

$$x_{\delta \mu_{a}}(\lambda_{i})=W(\lambda_{i})y_{\Delta \mathrm{OD}}(\lambda_{i}),$$ (2)

where $W(\lambda_{i})$ is the pseudo-inverse of $A(\lambda_{i})$ ($n_{\text{vertices}}$ by $n_{\text{channels}}$). We discuss below how to get this pseudo-inverse.

If the absorption is assumed to be dominated by hemoglobin, then $x_{\delta \mu_{a}}(\lambda_{i})$ for a given wavelength can be expressed as:

$$x_{\delta \mu_{a}}(\lambda_{i})=\epsilon_{\mathrm{HbO}}(\lambda_{i})x_{\Delta \mathrm{HbO}}+\epsilon_{\mathrm{HbR}}(\lambda_{i})x_{\Delta \mathrm{HbR}},$$ (3)

where ϵ is the molar extinction coefficient of HbO and HbR and is wavelength dependent. Using two wavelengths will give a unique solution for $x_{\Delta \mathrm{HbO}}$ and $x_{\Delta \mathrm{HbR}}$:

$$\left[\begin{array}{c} x_{\Delta \mathrm{HbO}}\\ x_{\Delta \mathrm{HbR}} \end{array}]=[\begin{array}{cc} \epsilon_{\mathrm{HbO}}(\lambda_{1}) & \epsilon_{\mathrm{HbR}}(\lambda_{1})\\ \epsilon_{\mathrm{HbO}}(\lambda_{2}) & \epsilon_{\mathrm{HbR}}(\lambda_{2}) \end{array}{]}^{-1}[\begin{array}{c} x_{\delta \mu_{a}}(\lambda_{1})\\ x_{\delta \mu_{a}}(\lambda_{2}) \end{array}\right]$$ (4)

We refer to this approach as the indirect method for reconstructing images of the changes in the hemoglobin concentrations and the pseudo-inverse as $W_{\text{indirect}}$. It is possible to combine Eqs. (2) and (4) so that the images of the changes in the hemoglobin concentration can be directly reconstructed from the changes in optical density at the two wavelengths in a single step. Specifically, as described in Ref. 31:

$$[\begin{array}{c} y_{\Delta \mathrm{OD}}(\lambda_{1})\\ y_{\Delta \mathrm{OD}}(\lambda_{2}) \end{array}]=[\begin{array}{cc} A(\lambda_{1}) & 0\\ 0 & A(\lambda_{2}) \end{array}][\begin{array}{c} x_{\delta \mu_{a}}(\lambda_{1})\\ x_{\delta \mu_{a}}(\lambda_{2}) \end{array}],$$ (5a)

$$[\begin{array}{c} y_{\Delta \mathrm{OD}}(\lambda_{1})\\ y_{\Delta \mathrm{OD}}(\lambda_{2}) \end{array}]=[\begin{array}{cc} \epsilon_{\mathrm{HbO}}(\lambda_{1})A(\lambda_{1}) & \epsilon_{\mathrm{HbR}}(\lambda_{1})A(\lambda_{1})\\ \epsilon_{\mathrm{HbO}}(\lambda_{2})A(\lambda_{2}) & \epsilon_{\mathrm{HbR}}(\lambda_{2})A(\lambda_{2}) \end{array}][\begin{array}{c} x_{\Delta \mathrm{HbO}}\\ x_{\Delta \mathrm{HbR}} \end{array}].$$ (5b)

which gives the following inverse problem, which will be referred to as the direct method:

$$[\begin{array}{c} x_{\Delta \mathrm{HbO}}\\ x_{\Delta \mathrm{HbR}} \end{array}]=W_{\text{direct}}[\begin{array}{c} y_{\Delta \mathrm{OD}}(\lambda_{1})\\ y_{\Delta \mathrm{OD}}(\lambda_{2}) \end{array}],$$ (6)

where $W_{\text{direct}}$ has dimensions $2*n_{\text{vertices}}$ by $2*n_{\text{channels}}$. All image space quantities for ΔHbO and ΔHbR are expressed in units of molar concentration change, M. In both cases (direct and indirect), the inverse problem is ill-posed and underdetermined. Several regularization strategies can be employed to help optimize the image reconstruction.

#### 2.1.1 Getting the inverse operator

As denoted above, W is the pseudoinverse of the sensitivity matrix A. The formulation for this inverse operator can be derived using Bayesian estimation, which aims to maximize the probability of the image space results, X, given the measurements in channel space, y. The expression for this probability can be written using Bayes’ rule:

$$P(X|y)=\frac{P(y|X)P(X)}{P(y)},$$ (7)

where P(X|y) is termed the posterior, the probability of the image given the measurements; P(y|X) is termed the likelihood, the probability of the measurements given the image; P(X) is termed the prior, the probability of the image; and P(y) measures how well the model (likelihood + prior) explains the observed data overall.

The expression for the model of the measurements, y, can be written using the forward model plus a noise term:

y=AX+n. (8)

If n and X are assumed to be normally distributed with zero mean and covariance matrices C and R, respectively, then Bayes’ rule gives expressions for P(y|X) and P(X)^38^:

$$P(y|X)\propto e^{-(AX-y{)}^{T}C^{-1}(AX-y)},$$ (9a)

$$P(X)\propto e^{-X^{T}R^{-1}X}.$$ (9b)

This allows us to derive the maximum of the posterior probability, max[P(X|y)], and obtain an expression for the pseudoinverse as:

$$W=(A^{T}C^{-1}A+R^{-1}{)}^{-1}A^{T}C^{-1}.$$ (10)

(See Ref. 38 for the full derivation.) When used in the inverse problem, X=Wy, this expression of W will yield the image that maximizes the posterior probability. Using the Bayesian formulation allows us to assign physiological meaning to both R and C.

The pseudoinverse can also be written in the form:

$$W={RA}^{T}({ARA}^{T}+C{)}^{-1}.$$ (11)

This is widely used and is shown to be equivalent to Eq. (10) in the work of Ref. 38. Due to their equivalence, we use Eq. (11) because the matrix inversion has dimension measurements by measurements instead of vertices (or voxels) by vertices (or voxels), which reduces the computational load and memory requirements.

#### 2.1.2 Choosing appropriate priors

As mentioned above, the Bayesian framework assigns meaning to the matrices R and C. The matrix, C, is considered the covariance of the measurement noise,^38^ which can be estimated directly from the data and is discussed further in Secs. 2.3.2 and 2.5.1. Conversely, R is the covariance of the image and cannot be estimated directly. It instead needs to be approximated using prior information and assumptions on the variance structure of the image. Here, we incorporate both expectations on the spatial and relative magnitude structure of the image covariance.

Spatial priors can be used to impose spatial constraints on the image reconstruction. Reconstructing images on the brain and scalp surfaces simultaneously has the benefit of enabling separation of brain signals from contaminating signals in the scalp.^12^^,^^39^^,^^40^ The measurements, however, have much higher sensitivity to the scalp than to the brain, and as a result, the joint image reconstruction can result in true brain activation signals being reconstructed in the scalp. Spatial regularization approaches have been developed that re-weight the sensitivity of the vertices to suppress the effect of the scalp and, as a result, control the depth of the reconstruction.^39^ As described in Ref. 40, this approach provides a prior for the image that suppresses image amplitude in the scalp and increases image amplitude in the brain. This prior is defined by the diagonal matrix L:

$$\lambda_{\text{spatial}}=\alpha_{\text{spatial}}\;\max(\mathrm{diag}(A^{T}A)),$$ (12)

$$\mathrm{diag}(L)=\sqrt{\mathrm{diag}(A^{T}A)+\lambda_{\text{spatial}}},$$ (13)

$$R=(L^{-1})(L^{-1}{)}^{T}.$$ (14)

This can then be scaled by a factor $\lambda_{R}$, which sets the expected magnitude of the image covariance. For the direct method, we set $\lambda_{R}$ to $10^{-6}$, which gives a maximum image standard deviation of $6*10^{-7}\;M$ for the range of $\lambda_{\text{spatial}}$ we utilized here. This is physiologically reasonable for the standard deviation of ΔHbO. For the indirect method, it is then possible to compute a value of $\lambda_{R}$ for each wavelength that yields an equivalent maximum image standard deviation for ΔHbO.

#### 2.1.3 Measurement regularization

As the inverse problem has significantly fewer observations than unknowns, the reconstruction is highly susceptible to noise. Furthermore, image reconstruction will attempt to resolve spatial patterns that are beyond the expected resolution of fNIRS. This means spatial smoothing can yield both less noisy and more accurate images. We start by regularizing the inverse based on the expected variance in the measurements, C, using the derivation of the inverse operator obtained by minimizing the expected error between the estimated and correct solution [extension of Eq. (11), see Ref. 38 for more details]:

$$W={RA}^{T}({ARA}^{T}+\lambda_{\mathrm{meas}}C{)}^{-1}.$$ (15)

Here, $\lambda_{\mathrm{meas}}$ is the regularization parameter, which we set as:

$$\lambda_{\mathrm{meas}}=\lambda_{R}\alpha_{\mathrm{meas}}\;\max(\mathrm{eig}({AA}^{T})),$$ (16)

where $\alpha_{\mathrm{meas}}$ is a scalar that can be varied to adjust the balance between image noise and spatial resolution. C can be chosen depending on how the measurement noise is estimated. In the standard approach, the identity matrix, I, is used under the assumption that measurement noise is the same across all channels, reducing Eq. (15) to the well-known Tikhonov Regularization approach used in DOT.^41^^,^^42^ As mentioned above, the measurement covariance can instead be estimated directly from real data and used in place of the identity matrix, as we describe in Secs. 2.3.2 and 2.5.1.

#### 2.1.4 Spatial basis functions

Finally, spatial basis functions can be employed to better model the spatial resolution expected with fNIRS and reduce the degrees of freedom of the inverse problem, which helps handle the ill-posed nature of the matrix inverse.^19^^,^^32^ We follow the method described in Ref. 19 where overlapping 3D Gaussian kernels are used on the surface of the brain and scalp to model hemodynamic changes at a given vertex:

$$g(x_{i})=\exp(-\frac{{\left\|x_{i}-x_{o}\right\|}^{2}}{2\sigma_{g}^{2}}),$$ (17)

where $x_{o}$ represents the center vertex of the Gaussian kernel, $x_{i}$ represents the location of another given vertex, and $\sigma_{g}^{2}$ is the standard deviation of the Gaussian, which controls the width of the kernel. The spatial bases are placed only on regions of the head with measurement sensitivity above a given threshold. In this case, only vertices with a summed measurement sensitivity greater than 0.01 were considered [calculated by summing the sensitivity matrix A(λ=850  nm) across channels].

The distance between the kernels is set equal to the standard deviation of the Gaussian, $\sigma_{g}$. Varying $\sigma_{g}$ of the brain and scalp impacts the spatial resolution, crosstalk between brain and scalp, and the smoothness of the images. The matrix G is a spatial basis matrix with dimensions number of vertices by number of spatial bases. Each column contains the spatial representation of a given spatial basis function on the surface of the brain and scalp.

For the indirect method, the forward model can then be rewritten such that $x_{\delta \mu_{a}}(\lambda_{i})$ is represented as the weighted sum of the spatial basis set, G, with coefficients b:

$$x_{\delta \mu_{a}}(\lambda_{i})=G(\lambda_{i})b(\lambda_{i}).$$ (18)

Thus, we have:

$$y_{\Delta \mathrm{OD}}(\lambda_{i})=A(\lambda_{i})G(\lambda_{i})b(\lambda_{i}).$$ (19)

By letting $H(\lambda_{i})=A(\lambda_{i})G(\lambda_{i})$, the pseudo-inverse [using Eq. (11)] can be used to get the weights of each spatial basis function:

$$b(\lambda_{i})=H^{-1}(\lambda_{i})y_{\Delta \mathrm{OD}}(\lambda_{i}).$$ (20)

The weights, $b(\lambda_{i})$, can then be transformed back to $x_{\delta \mu_{a}}(\lambda_{i})$ using Eq. (18) and converted to $x_{\Delta \mathrm{HbO}}$ and $x_{\Delta \mathrm{HbR}}$ using Eq. (4). The problem can be similarly setup for the direct method to obtain $x_{\Delta \mathrm{HbO}}$ and $x_{\Delta \mathrm{HbR}}$ as:

$$[\begin{array}{c} x_{\Delta \mathrm{HbO}}\\ x_{\Delta \mathrm{HbR}} \end{array}]=Gb,$$ (21)

$$[\begin{array}{c} y_{\Delta \mathrm{OD}}(\lambda_{1})\\ y_{\Delta \mathrm{OD}}(\lambda_{2}) \end{array}]=AGb.$$ (22)

The inverse problem can be handled as described above by getting the pseudoinverse of H=AG using Eq. (11), solving for the weights b:

$$b=H^{-1}[\begin{array}{c} y_{\Delta \mathrm{OD}}(\lambda_{1})\\ y_{\Delta \mathrm{OD}}(\lambda_{2}) \end{array}],$$ (23)

and finally, transforming the weights in spatial basis space back into concentration changes on the cortical surface using Eq. (21).

The optimization of this pipeline requires the appropriate selection of $\alpha_{\mathrm{meas}}$, $\alpha_{\text{spatial}}$, $\sigma_{\text{brain}}$, and $\sigma_{\text{scalp}}$ as well as a comparison between the indirect and direct methods to evaluate and minimize crosstalk between chromophores.

### 2.2 Data Collection

#### 2.2.1 Whole-head high-density probe design

**Fig. 1:**
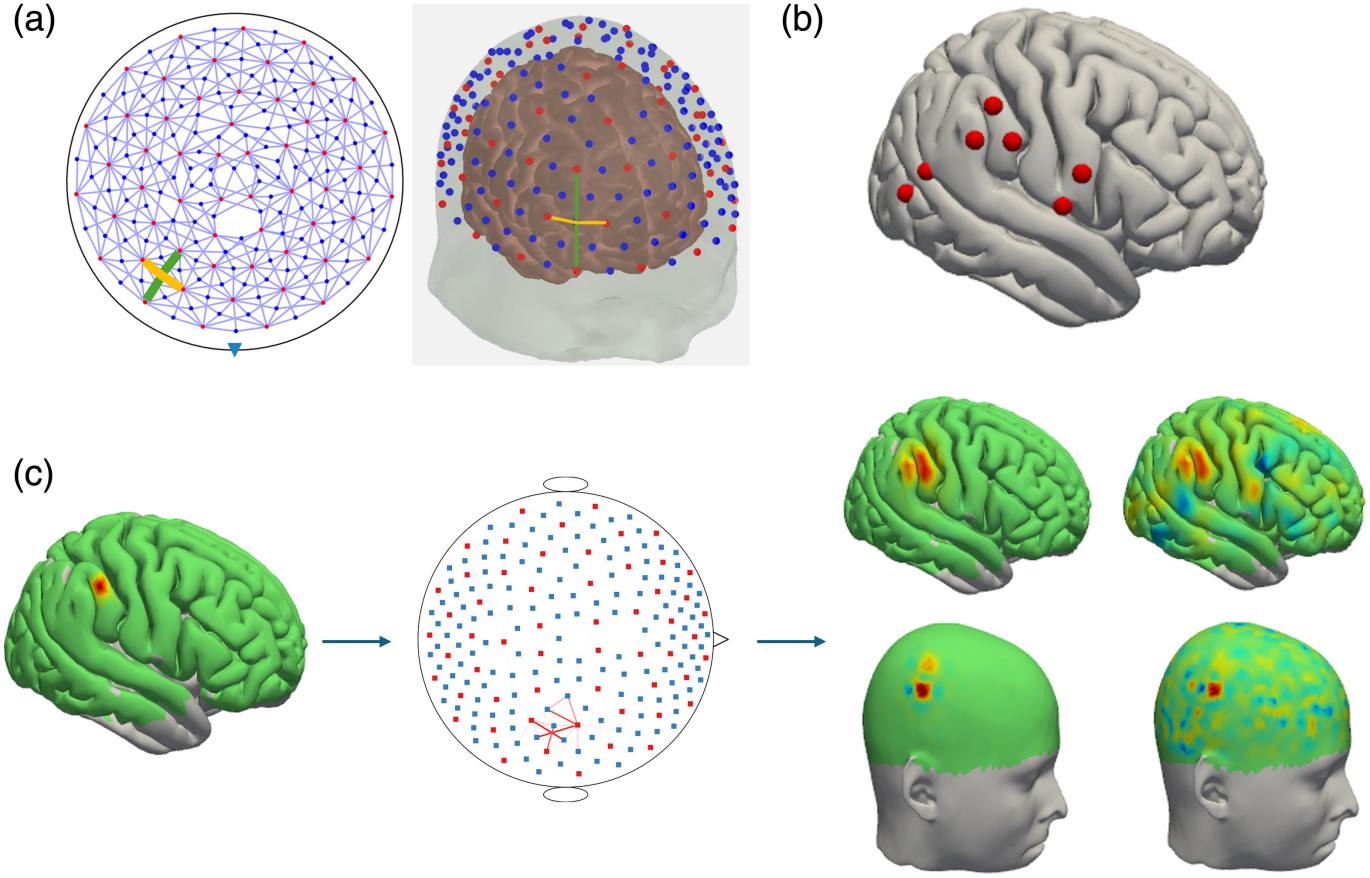
(a) NinjaNIRS2022 probe layout in both 2D (left) and on a 3D head model (right). Orange channel marking indicates the first nearest neighbor source-detector pairs at 19 mm and green channel marking indicates the second nearest neighbor source-detector pairs at 33 mm. (b) Head model showing the seed vertices used in all simulation studies as indicated with the red dot. (c) Overview of the simulation pipeline. Starts with a simulated Gaussian blob of activation (here σ=3  mm) (left). The Gaussian activation is projected into channels space using the forward model (middle). The optical density measurements in channel space are reconstructed on the brain and scalp surfaces (right). The leftmost images show a noise-free reconstruction, whereas the rightmost images show the reconstruction in the presence of white noise.

The collection and simulation of all data in this paper were done using a hexagonal whole-head, high-density probe layout [see Fig. 1(a)] developed for the NinjaNIRS2022 device.^27^ Whole head coverage was achieved using 56 sources and 144 detectors with first-nearest neighbor channels separated by 19 mm and second-nearest neighbor channels separated by 33 mm. This yields 567 measurement channels per wavelength. Data are collected at two wavelengths, 760 and 850 nm, giving 1134 total measurements.

#### 2.2.2 Study design

Data were collected from healthy adult subjects using a study protocol approved by the Institutional Review Board of Boston University. All participants provided written informed consent to take part in the study. The data from 17 participants were used in the study. The participant demographics were reported as follows: mean age = 29.5 ± 9.7 years; 7 females, 10 males; 17 right-handed; 11 White, 5 Asian, and 1 Black or African American.

All participants were asked to perform a 6-min resting state run. During this measurement, subjects sat in front of a computer screen and were instructed to remain as still as possible and remain mentally idle while fixing their gaze on a crosshair on the screen.

Participants were also asked to perform a ball-squeezing task. Participants were seated in front of a computer and prompted to squeeze the ball in either their left or right hand according to the prompt on the computer screen. Each trial was 5 s, and the rest in between was jittered from 15 to 20 s. Each run contained 14 total trials, resulting in seven trials each of the left- and right-handed task and each participant performed three runs. The order of the trials within a run was presented in a pseudorandom order and varied across runs. All task presentations were created using PsychoPy software.^43^ All pre-processing and forward modelling were done using the Cedalion Toolbox.^44^ Data are available on OpenNeuro.^45^

### 2.3 Simulation Study

The ICBM152 head model as implemented in the Cedalion toolbox^46^ and the Monte Carlo simulation from pmcx^36^ were used to generate the sensitivity profile for each wavelength. The brain surface contained 15,002 vertices, each controlling a median volume of $71\;{\mathrm{mm}}^{3}$. Similarly, the scalp surface contained 10,018 vertices, each controlling a median volume of $161\;{\mathrm{mm}}^{3}$. At each optode, $10^{8}$ photons were simulated. The absorption coefficients, $\mu_{a}$, and reduced scattering coefficients, $\mu_{s}^{\prime }$, that were used for each tissue type (scalp, skull, cerebral spinal fluid, white matter, gray matter) were taken from Ref. 19 and are listed in Table 1.

**Table 1:** Absorption and reduced scattering coefficients for different tissues.

Tissue	$\mu_{a}$ (${\mathrm{mm}}^{-1}$)		$\mu_{s}^{\prime }$ (${\mathrm{mm}}^{-1}$)	
	760 nm	850 nm	760 nm	850 nm
White matter	0.0195	0.0208	1.18	1.01
Grey matter	0.0195	0.0192	1.18	0.67
Skull	0.0125	0.0139	0.93	0.84
Scalp	0.0177	0.0190	0.73	0.64
Cerebral spinal fluid	0.0021	0.0040	0.30	0.30

#### 2.3.1 Regularization parameter optimization

We had four regularization parameters to optimize: $\alpha_{\mathrm{spatial}}$, $\alpha_{\mathrm{meas}}$, $\sigma_{\text{brain}}$, and $\sigma_{\text{scalp}}$.

##### Selecting $\alpha_{\text{spatial}}$

To help determine the optimal parameter for $\alpha_{\text{spatial}}$, we utilized the singular value spectrum of ${\mathrm{AA}}^{T}$. The singular value spectrum gives insight into the stability of the inverse of A as well as insight into which spatial frequencies can be reliably reconstructed. Small singular values tend to be associated with image eigenvectors representing high-frequency spatial patterns beyond the expected spatial resolution of fNIRS.^15^^,^^20^^,^^40^ When A is inverted, these eigenvectors will be amplified, which will lead to noisy images. Regularization modifies the matrix that is to be inverted, generally resulting in a singular value spectrum that decays less fast. As such, we aim to maximize the sum of the singular values to guide the selection of $\alpha_{\text{spatial}}$. We do this by first normalizing each spectrum such that the maximum singular value is one and then taking the resulting area under the curve (AUC).

##### Image metrics

To select the value of $\sigma_{\text{brain}}$, $\sigma_{\text{scalp}}$, and $\alpha_{\mathrm{meas}}$, a simulation study was performed to evaluate the impact of these parameters on several image quality metrics outlined in Table 2: image full-width half-maximum (FWHM), contrast-to-noise ratio (CNR), localization error, brain-to-scalp crosstalk, percent signal predicted by the brain, and the crosstalk between ΔHbO and ΔHbR. For the simulations involving a single wavelength, a Gaussian blob of activation-induced absorption changes at 850 nm was modelled with a spatial standard deviation of 3 mm centered at a given seed vertex on the brain surface (note that this corresponds to a FWHM of 2.35*3  mm=6.9  mm). No activation was put on the scalp surface. The forward model described in Eq. (1) was used to project this ground truth image into channel space. The maximum amplitude of the absorption change was set such that a maximum change in optical density of 0.02 was measured on the channel with maximum sensitivity to the absorption change. We centered this absorption change at seven different seed locations as indicated in Fig. 1(b) for the simulation study. The seeds were selected such that we spatially sampled vertices based both on their placement relative to optodes on the probe and their location either on a gyrus or sulcus. The Gaussian blob for one of these locations and the resulting ΔOD in channel space is presented in Fig. 1(c).

**Table 2:** Overview of image quality metrics.

Metric	Definition
ROI brain	⇒ All vertices with amplitude greater than 0.5 times the maximum amplitude of the noise-free reconstructed image of the brain
ROI scalp	⇒ All vertices with amplitude greater than 0.5 times the maximum amplitude of the noise-free reconstructed image of the scalp
Single wavelength	
Full width half maximum (FWHM)	⇒ Weighted mean distance between the centroid of the ROI and all other vertices in the ROI—weights come from the image amplitude. This weighted mean is then scaled by 2.35 to obtain the FWHM. This is an accurate estimate of the FWHM for a Gaussian image and an approximation for images with other shapes.
Contrast-to-noise ratio (CNR)	⇒ Contrast given by the mean of the noise-free image within the ROI
	⇒ Noise—for 100 noise instances
	⇒ Noise from a Gaussian distribution with μ=0 and σ=0.01 was added to all measurements
	⇒ Reconstruct the noisy image
	⇒ Take standard deviation of the means across all noise instances
	⇒ Divide the contrast by the noise
Contrast ratio	⇒ The ratio between the maximum of the reconstructed image and the maximum of the ground truth image
Localization error	⇒ Euclidean distance between the centroid of the ROI and the center of the original image
Crosstalk brain → scalp	⇒ Mean of the noise-free image within the ROI of the brain divided by the mean of the noise-free image within the ROI of the scalp
Percent signal predicted by brain	⇒ The noise-free brain image is projected back to channel space using the forward model to get measurements predicted by brain
	⇒ Divide the sum of the measurements predicted by the brain by the ground truth measurements
Dual wavelength	
Crosstalk ΔHbO → ΔHbR	⇒ Start with a ground truth image of only ΔHbO activation
	⇒ Define ROI using ΔHbO image reconstruction
	⇒ Mean of the ROI of the ΔHbR image divided by the mean of the ROI of the ΔHbO image
Crosstalk ΔHbR -> ΔHbO	⇒ Start with a ground truth image of only ΔHbR activation
	⇒ Define ROI using ΔHbR image reconstruction
	⇒ Mean of the ROI of the ΔHbO image divided by the mean of the ROI of the ΔHbR image

The images were then reconstructed from the 850 nm optical density measurements using varying $\alpha_{\mathrm{meas}}$, $\sigma_{\text{brain}}$, and $\sigma_{\text{scalp}}$. The identity matrix was used as the measurement covariance when calculating the pseudoinverse, W, in Eq. (11). An example noise-free image for a given set of regularization parameters is shown in Fig. 1(c). The single wavelength metrics in Table 2 were calculated for each set of parameters at each seed vertex. For all metrics, a region of interest (ROI) is defined as all the vertices with an amplitude greater than 0.5 times the maximum amplitude of the noise-free reconstructed image. The mean and standard error across the seven different seed locations for each metric are reported for each parameter combination.

For the dual wavelength metrics, a Gaussian blob of activation-induced concentration changes at either ΔHbO or ΔHbR was modelled with a standard deviation of 3 mm centered at a given seed vertex while the image of the other chromophore is set to zero. The forward model described in Eq. (5b) was used to project the ground truth images to channel space, resulting in optical density measurements for both 760 and 850 nm. The optical density measurements for both wavelengths in channel space were scaled relatively so that the maximum amplitude for the optical density change in 850 nm was 0.02 on the channel with maximum sensitivity to the absorption change.

Images were reconstructed from the optical density measurements using both the direct and indirect methods with varying $\alpha_{\mathrm{meas}}$, $\sigma_{\text{brain}}$, and $\sigma_{\text{scalp}}$. The calculation of crosstalk for ΔHbO into ΔHbR, and ΔHbR into ΔHbO was calculated depending on which chromophore was used as the ground truth activation. Further metrics were calculated when the ground truth was an activation in ΔHbO. The reconstructed ΔHbO image was used to obtain CNR, FWHM, localization error, and crosstalk from the brain into the scalp. The mean and standard error across the seven different seed locations for each metric are reported for each parameter combination for both the direct and indirect methods.

#### 2.3.2 Parameter selection validation using an augmented dataset

##### Data augmentation

The selection of parameters chosen using the simulation study was further refined using real measurement noise to define the measurement covariance in Eq. (11) to confirm the selection of $\alpha_{\mathrm{meas}}$. First, $C_{\mathrm{meas}}$ was estimated by augmenting resting-state data collected during a 6 min run from 17 subjects using functionality from Cedalion. A synthetic HRF was defined for ΔHbO and ΔHbR using a single gamma function with the following parameters: τ=0.1 and 1.8 s for ΔHbO and ΔHbR, respectively, and σ=3  s for both chromophores.^32^ Each gamma function was convolved with a 5 s boxcar function to model a 5 s stimulus. The magnitude of the synthetic HRF being added per channel was based on a Gaussian blob of activation-induced absorption changes with a spatial standard deviation of 15 mm centered at a given seed vertex, which is projected to channel space using the forward model. The maximum channel was set to be a 0.02 change in optical density at 850 nm. The HRF was added to the resting-state optical density timeseries at jittered, nonoverlapping intervals between 10 and 20 s.

The augmented timeseries was minimally pre-processed. The measured intensity was converted to ΔOD and then to concentration using the modified Beer–Lambert law. A general linear model (GLM) was used to estimate the HRF using consecutive Gaussian functions with a standard deviation of 1 s and 1 s spacing to model the response from 2 s before to 16 s after the onset of each trial.^47^ The GLM also included mid-separation regression using the average of all the 19 mm channels and drift correction using third-order Legendre polynomials.^48^^,^^49^ The GLM was solved using the autoregressive iterative reduced least squares (AR-IRLSs) approach outlined in Ref. 50. This approach iteratively fits the GLM, estimates temporal autocorrelation in the residuals using an autoregressive (AR) model, and applies pre-whitening to both the data and design matrix. This procedure accounts for serially correlated noise and yields unbiased parameter estimates and more accurate variance estimates. The results yield the estimate of the timeseries for the HRF and within-subject covariance. For simplification, we took only the diagonal of the covariance matrix and set all the off-diagonal terms to zero.

We denote this within-subject variance as $C_{\mathrm{meas}}$ and took the mean of $C_{\mathrm{meas}}$ over the temporal window between 5 and 8 s. This represents the peak of the HRF response and provided a single measure of variance for each channel and each chromophore. These variances were then converted back to optical density variances to be included as the measurement regularization term in the image reconstruction.

##### Image metrics

First, for each of the seven seed vertices, noise-free ground truth images were generated as described in Sec. 2.3.1 by modelling a Gaussian blob of activation-induced absorption changes at 850 nm with a standard deviation of 15 mm to match the activation used when estimating $C_{\mathrm{meas}}$. This was projected into channel space and scaled such that the channel with maximum sensitivity to the absorption change had a change in optical density of 0.02. An image was reconstructed from the noise-free data for each subject using the within-subject variance for optical density in 850 nm ($C_{\mathrm{meas}}$) for the measurement covariance in the pseudoinverse, W [Eq. (11)]. An image of the within-subject variance, $C_{\text{image}}$, was also generated by propagating $C_{\mathrm{meas}}$ into image space, as outlined in Sec. 2.5.2.

For each seed vertex, the group mean, $X_{\text{mean}}$, and the total variance, $\sigma_{\mathrm{tot}}^{2}$, were estimated across subjects using an iterative approach (more details in Sec. 2.5.3.). The final group average estimate is used to obtain the single-wavelength image metrics in Table 2. For CNR, however, the total variance can be used directly as the noise estimate and becomes $\mathrm{CNR}=\frac{X_{m\mathrm{ean}}}{\sigma_{\mathrm{to}t}}$. The image metrics are calculated at each seed vertex, and the results are reported as the mean and standard error across vertices.

For the dual-wavelength metrics, the approach is again like in Sec. 2.3.1. A Gaussian blob of activation-induced concentration changes at either ΔHbO or ΔHbR was modelled with a standard deviation of 15 mm centered at a given seed vertex while the image of the other chromophore is set to zero. The forward model described in Eq. (5b) was used to project the ground truth images to channel space, resulting in optical density measurements for both 760 and 850 nm. The optical density measurements for both wavelengths in channel space were scaled relatively so that the maximum channel for 850 nm had a change in optical density of 0.02. Using both the direct and indirect methods, noise-free images of ΔHbO and ΔHbR were reconstructed for each subject using the within-subject variance ($C_{\mathrm{meas}}$) for the measurement covariance in the pseudoinverse, W [Eq. (11)]. The image of the within-subject variance, $C_{\text{image}}$, was also generated by propagating $C_{\mathrm{meas}}$ into image space as outlined in Sec. 2.5.2.

For each seed vertex, the group means and total variance were estimated across subjects using the iterative approach outlined in Sec. 2.5.3. The group mean was used to obtain the image metrics outlined in Table 2. The crosstalk of ΔHbO into ΔHbR and ΔHbR into ΔHbO was calculated depending on which chromophore was used as the ground truth activation. Further metrics were calculated when the ground truth was an activation in ΔHbO. The reconstructed group mean ΔHbO image was used to obtain CNR, FWHM, localization error, and crosstalk from the brain into the scalp. The mean and standard error across the seven different seed locations for each metric are reported for each parameter combination for both the direct and indirect methods. The code required to perform the data augmentation and calculate the image quality metrics has been made publicly available on GitHub (see Code and Data Availability).

### 2.4 Ball-Squeezing Task

#### 2.4.1 Data processing

The ball-squeezing data from 17 subjects were preprocessed using the Cedalion toolbox as follows. The intensity was converted to optical density and then converted to concentration. As described above, a general linear model (GLM) was used to estimate the HRF using consecutive gamma functions with a standard deviation of 1 s and 1 s spacing to model the response from 2 s before to 15 s after the onset of each trial.^47^ The GLM included mid-separation regression using the average of all the 19 mm channels and drift correction using third-order Legendre polynomials^48^^,^^49^ and was solved using the autoregressive model outlined in Ref. 50 and described in more detail in Sec. 2.3.2. This provided the estimate of the timeseries for the HRF magnitude and within-subject covariance. We again only kept the diagonal of the covariance matrix and set the off-diagonal terms to zero to simplify downstream computations. This resulted in the diagonal within-subject variance matrix, $C_{\mathrm{meas}}$, for each time point of the HRF.

The estimated HRF and $C_{\mathrm{meas}}$ for each subject and trial type were averaged over the window from 5 to 8 s and converted back to optical density. This magnitude was projected into image space, and the error was propagated (Sec. 2.5.2) to get the magnitude images, $x_{\mathrm{HbX}}$ and covariance images, $C_{\text{image},\Delta \mathrm{HbX}}$. The group average and total variance were obtained as per Eqs. (33) and (36) (Sec. 2.5.3), respectively, and used to calculate the t-statistic.

To obtain the image space timeseries of the full HRF, the HRF timeseries for each subject was projected into image space. As the measurement covariance varies over time, in this case, $C_{\mathrm{meas}}$ was averaged over the full timeseries and used in Eq. (11).

The group-averaged ΔHbO magnitude was used to define an ROI. All vertices with an amplitude greater than 50% of the max amplitude were selected. We then calculated the variance-weighted HRF across the ROI for each subject using $C_{\text{image},\Delta \mathrm{HbX}}$:

$$x_{\mathrm{ROI},\Delta \mathrm{HbX}}(t)=\frac{\Sigma_{v=1}^{n_{\text{vertex},\mathrm{ROI}}}w_{v}x_{\Delta \mathrm{HbX},v}(t)}{\Sigma_{v=1}^{n_{\text{vertex},\mathrm{ROI}}}w_{v}},$$ (24)

where the weights are given as $w_{v}=\frac{1}{C_{\text{image},\Delta \mathrm{HbX}}(v)}$ for vertex v belonging to the ROI with $n_{\text{vertex},\mathrm{ROI}}$ total vertices. The timeseries $x_{\mathrm{ROI},\Delta \mathrm{HbX}}(t)$ can be obtained for both ΔHbX=ΔHbO and ΔHbR using their respective $C_{\text{image},\Delta \mathrm{HbX}}$. The within-subject variance for the ROI can also be obtained from the weights using:

$$C_{\mathrm{ROI},\Delta \mathrm{HbX}}=\frac{1}{\Sigma_{v=1}^{n_{\text{vertex},\mathrm{ROI}}}w_{v}}.$$ (25)

From here, the group average and total variance for the timeseries within the ROI can be obtained following the framework outlined in Sec. 2.5.3.

### 2.5 Estimating Covariances and Group Average

#### 2.5.1 Within-subject covariance

The within-subject variance matrix estimated from AR-IRLS (Sec. 2.3.2) has off-diagonal terms that we set to zero. As mentioned above, we are only utilizing the diagonal of this covariance matrix to simplify downstream computation:

$${\overset{˜}{C}}_{\mathrm{meas}}(\lambda_{i})=[\;\begin{array}{cccc} \sigma_{1}^{2}(\lambda_{i}) & 0 & \cdots & 0\\ 0 & \sigma_{2}^{2}(\lambda_{i}) & \cdots & 0\\ \vdots & \vdots & \ddots & \vdots \\ 0 & 0 & \cdots & \sigma_{N}^{2}(\lambda_{i}) \end{array}],$$ (26)

where ${\overset{˜}{C}}_{\mathrm{meas}}(\lambda_{i})$ is the measurement covariance matrix for a single wavelength, $\lambda_{i}$, and $\sigma_{j}^{2}(\lambda_{i})$ is the variance for a given channel, j=1…N (number of channels). The measurement covariance matrices for both wavelengths can be stacked to give the diagonal matrix:

$$C_{\mathrm{meas}}=[\begin{array}{cc} {\overset{˜}{C}}_{\mathrm{meas}}(\lambda_{1}) & O\\ O & {\overset{˜}{C}}_{\mathrm{meas}}(\lambda_{2}) \end{array}].$$ (27)

For channels that were either saturated or had a mean amplitude close to the noise floor, their variance will be low despite the channel containing bad data. The entry for these channels was manually set to a high value to indicate that the data in this channel were not trusted. We selected 10 for this high value. A minimum value threshold was used to prevent channels with variance significantly lower than the mean from being overly trusted. We choose the threshold by looking at the distribution of variances from all subjects and choosing the value to the left of the peak at which the histogram was ∼25% of the peak value. In our case, this corresponded to a minimum value threshold of $10^{-6}$.

As mentioned above, in Eq. (15), the identity matrix can be used to represent a uniform measurement variance across channels. However, to get a more accurate estimate of the measurement noise, the measurement covariance, $C_{\mathrm{meas}}$, can instead be used.

#### 2.5.2 Error propagation to image space

The within-subject variance in image space was estimated using the posterior covariance. In the Bayesian framework, this is expressed as:

$$\Sigma_{X|y}=(R^{-1}+A^{T}C^{-1}A{)}^{-1},$$ (28)

or equivalently, using the Sherman–Morrison–Woodbury formula:

$$\Sigma_{X|y}=R-{RA}^{T}({ARA}^{T}+C{)}^{-1}AR.$$ (29)

For the direct method, this can be done in a single step where A takes the form:

$$A_{\text{direct}}=[\begin{array}{cc} \epsilon_{\mathrm{HbO}}(\lambda_{1})A(\lambda_{1}) & \epsilon_{\mathrm{HbR}}(\lambda_{1})A(\lambda_{1})\\ \epsilon_{\mathrm{HbO}}(\lambda_{2})A(\lambda_{2}) & \epsilon_{\mathrm{HbR}}(\lambda_{2})A(\lambda_{2}) \end{array}].$$ (30)

Here, R is defined using $A_{\text{direct}}$ and C is the within-subject measurement defined above, $C_{\mathrm{meas}}$. This returns $C_{\text{image},\text{direct}}$, which is the posterior covariance of the image with entries representing the variance for a given vertex in units of concentration.

For the indirect method, this is done in two steps. The posterior covariance can be estimated for each wavelength independently:

$$\Sigma_{X|y}(\lambda_{i})=R(\lambda_{i})-R(\lambda_{i})A(\lambda_{i}{)}^{T}(A(\lambda_{i})R(\lambda_{i})A(\lambda_{i}{)}^{T}+{\overset{˜}{C}}_{\mathrm{meas}}(\lambda_{i}){)}^{-1}A(\lambda_{i})R(\lambda_{i}).$$ (31)

This returns ${\overset{˜}{C}}_{\text{image},\;\delta \mu_{a}}(\lambda_{i})$. To convert this into units of concentration, the molar extinction coefficients are used, $\varepsilon =[\begin{array}{cc} \epsilon_{\mathrm{HbO}}(\lambda_{1}) & \epsilon_{\mathrm{HbR}}(\lambda_{1})\\ \epsilon_{\mathrm{HbO}}(\lambda_{2}) & \epsilon_{\mathrm{HbR}}(\lambda_{2}) \end{array}]$. We can then convert ${\overset{˜}{C}}_{\text{image},\delta \mu_{a}}(\lambda_{i})$ for a given vertex, j, into concentration, $C_{\text{image},\text{indirect},j}$, by doing the following:

$$C_{\text{image},\text{indirect},j}=\varepsilon [\begin{array}{cc} C_{\text{image},\delta \mu_{a},j}(\lambda_{1}) & 0\\ 0 & C_{\text{image},\delta \mu_{a},j}(\lambda_{2}) \end{array}]\varepsilon^{T}.$$ (32)

where $C_{\text{image},\delta \mu_{a},j}(\lambda_{i})$ is the scalar covariance at vertex, j, for a given wavelength, $\lambda_{i}$, and $C_{\text{image},\text{indirect},j}$ is the corresponding 2 by 2 covariance matrix in concentration. Here again, we take only the diagonal to get the variance of ΔHbO and ΔHbR for each vertex while ignoring the off-diagonal terms. We leave the investigation of these covariance terms for future work.

#### 2.5.3 Group average and total variance estimation

The group average image was obtained through an iterative process. First, following Ref. 34, a weighted group average was calculated using the within-subject variance as the weights. For a given subject i, the weights are given as, $w_{i}=\frac{1}{C_{\text{image},i}}$, and the initial estimation of the group mean can be expressed as:

$$x_{\text{mean}}=\frac{\Sigma_{i=1}^{n_{\text{sub}j}}w_{i}x_{i}}{\Sigma_{i=1}^{n_{\text{sub}j}}w_{i}}.$$ (33)

Here, $x_{i}$ is the mean HRF magnitude across epochs for subject i, and multiplication by the weights refers to elementwise multiplication per vertex. The mean within-subject variance is defined as ${\overset{¯}{\sigma }}_{\text{within}}^{2}$:

$${\overset{¯}{\sigma }}_{\text{within}}^{2}=\frac{1}{\Sigma_{i=1}^{n_{\text{sub}j}}w_{i}}.$$ (34)

The between-subject estimation, $\sigma_{\text{between}}^{2}$, is calculated using the initial group mean estimation in the following equation:

$$\sigma_{\text{between}}^{2}=\frac{1}{n_{\text{sub}j}}(\Sigma_{i=1}^{n_{\text{sub}j}}\frac{(x_{\text{mean}}-x_{i}{)}^{2}}{C_{\text{image},i}}){\overset{¯}{\sigma }}_{\text{within}}^{2}.$$ (35)

In the second iteration, the group mean is re-estimated using now both the within- and between-subject variances as the weights. The total variance can be expressed as $\sigma_{\mathrm{tot},i}^{2}=\sigma_{\text{between}}^{2}+C_{\text{image},i}$, so the corresponding weight becomes $w_{i}=\frac{1}{\sigma_{\mathrm{tot},i}^{2}}$. The final group mean is then calculated using Eq. (33) and the updated weights. The total variance can be calculated as:

$$\sigma_{\mathrm{tot}}^{2}=\frac{1}{\Sigma_{i=1}^{n_{\text{sub}j}}\frac{1}{\sigma_{\text{between}}^{2}+C_{\text{image},i}}}.$$ (36)

This can then be used along with the final estimate of the group mean to obtain the t-statistics.

#### 2.5.4 Considerations for the selection of $\lambda_{R}$ and $\alpha_{\mathrm{meas}}$

In this work, we have introduced an additional regularization parameter $\lambda_{R}$ to be used in the Bayesian formulation of the pseudoinverse. When this is used to generate the contrast images, $\lambda_{R}$ can be factored out to yield the traditional Tikhonov formulation where there is only regularization on the measurements:

$$W=\lambda_{R}{RA}^{T}(\lambda_{R}{ARA}^{T}+\lambda_{R}\alpha_{\mathrm{meas}}\;\max(\mathrm{eig}({ARA}^{T})C{)}^{-1},$$ (37)

$$W={RA}^{T}({ARA}^{T}+\alpha_{\mathrm{meas}}\;\max(\mathrm{eig}({ARA}^{T})C{)}^{-1}.$$ (38)

That said, the Bayesian formulation implies that there should be no scaling of the measurement covariance because this should already be scaled appropriately to represent the true measurement noise in the data. As we are obtaining C directly from the data, this is an estimate of the true measurement covariance. On the other hand, scaling R is valid because this needs to be tuned to the appropriate amplitude expected of the image covariance. This is of particular importance in our estimate of the posterior covariance because $\lambda_{R}$ cannot be factored out completely:

$$\Sigma_{X|y}=\lambda_{R}R-\lambda_{R}{RA}^{T}(\lambda_{R}{ARA}^{T}+\lambda_{R}\alpha_{\mathrm{meas}}\;\max(\mathrm{eig}({ARA}^{T})C{)}^{-1}AR\lambda_{R},$$ (39)

$$\Sigma_{X|y}=\lambda_{R}(R-{RA}^{T}({ARA}^{T}+\alpha_{\mathrm{meas}}\;\max(\mathrm{eig}({ARA}^{T})C{)}^{-1}AR).$$ (40)

For this to be a valid estimation of the posterior covariance, $\alpha_{\mathrm{meas}}$ should be selected such that $\lambda_{\mathrm{meas}}=\lambda_{R}\alpha_{\mathrm{meas}}\;\max(\mathrm{eig}({ARA}^{T})=1$ (where $\lambda_{R}$ is selected to be physiologically reasonable as described in Sec. 2.1.2). This corresponds in our case to an $\alpha_{\mathrm{meas}}$ on the order of $10^{4}$.

For the pure simulation, the identity matrix was used in place of C, and noise was artificially simulated, meaning the results have no dependence on the posterior covariance. As such, the selection of $\lambda_{R}$ has no bearing on the results from this portion of the study. For the augmented resting-state simulation and the ball-squeezing results, however, true measurement covariance is used as $C_{\mathrm{meas}}$, and the posterior covariance is used to estimate CNR and t-statistics, respectively. We continued to sweep $\alpha_{\mathrm{meas}}$ to assess the impact on the image metrics; however, we emphasize that only those with an $\alpha_{\mathrm{meas}}$ of $10^{4}$ are valid because this is the value where we can trust our estimate of the posterior covariance.

## 3 Results

### 3.1 Simulation Study

#### 3.1.1 Regularization parameter selection

##### Selecting $\alpha_{\text{spatial}}$

**Fig. 2:**
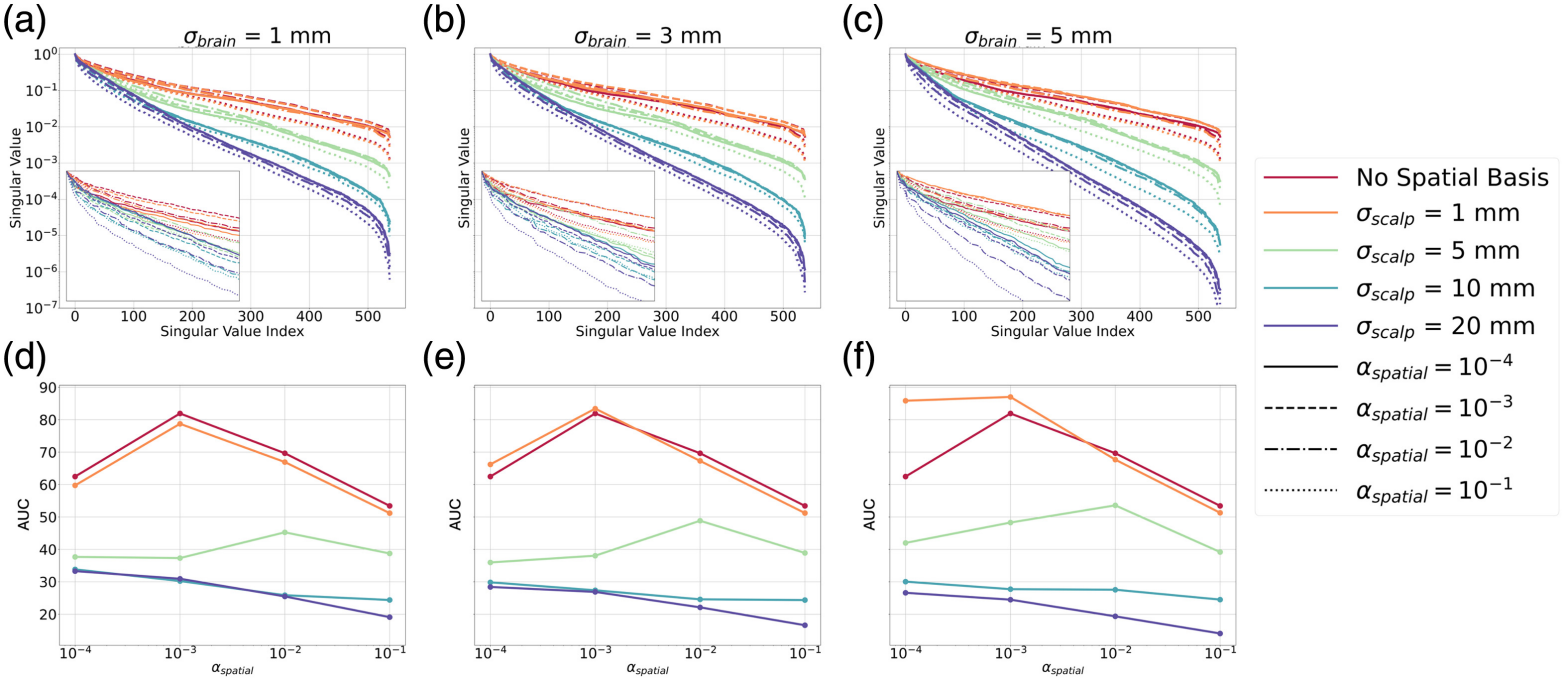
(a)–(c) Singular value spectra of A^A^T normalized such that the maximum singular value is 1. $\sigma_{\text{brain}}$ of 1 mm (a), 3 mm (b), and 5 mm (c). Each panel shows the case when no spatial basis functions are used in red. Varying color corresponds to varying $\sigma_{\text{scalp}}$. Varying line style corresponds to varying $\alpha_{\text{spatial}}$. The inset in the bottom left of each panel shows the zoomed-in region corresponding to the first 50 singular values. (d)–(f) AUC of each singular value spectrum versus the value of $\alpha_{\text{spatial}}$. The colors correspond to the same varying $\sigma_{\text{scalp}}$ as used in the top row. The case where no spatial basis functions are used is again shown in each panel in red. A peak in the AUC when no spatial basis functions are used is identified at $10^{-3}$, indicating that this is the optimal value of $\alpha_{\text{spatial}}$. This is also true when $\sigma_{\text{scalp}}$ is 1 mm across all values of $\sigma_{\text{brain}}$. When $\sigma_{\text{scalp}}$ is 5 mm, the peak is located at $10^{-2}$ across all values of $\sigma_{\text{brain}}$. For larger values of $\sigma_{\text{scalp}}$, there is no peak value for $\alpha_{\text{spatial}}$. Instead, there is a general decrease in the AUC as $\alpha_{\text{spatial}}$ increases.

The selection of $\alpha_{\text{spatial}}$ was based on the AUC of the normalized singular value spectrum of Fig. 2, which shows the singular value spectra of A^A^T for varying $\sigma_{\text{brain}}$ and $\sigma_{\text{scalp}}$, as $\alpha_{\text{spatial}}$ is swept from $10^{-4}$ to $10^{-1}$. The eigenvectors of A^A^T represent the spatial patterns that can be reconstructed on the brain and scalp surfaces. Their associated singular values indicate the reliability with which we can resolve those spatial patterns. Singular values that are small are often associated with higher-frequency spatial patterns, which then become amplified when the pseudoinverse is taken. This leads to noisier images. A singular value spectrum that decays more slowly and has a higher AUC will result in better quality image reconstructions and correspond to a better selection of $\alpha_{\text{spatial}}$.

Figures 2(d)–2(f) show that across all values of $\alpha_{\text{spatial}}$, the largest AUC is given when either $\sigma_{\text{scalp}}$ is 1 mm or when no spatial bases are used. As $\sigma_{\text{scalp}}$ increases, the number of spatial bases for reconstructing the image decreases, resulting in a faster decay of the singular value spectrum. The increase of $\sigma_{\text{brain}}$ does not have as large of an impact as $\sigma_{\text{scalp}}$ because the spatial resolution we can achieve on the brain is on a similar order of magnitude as the $\sigma_{\text{brain}}$ values we investigate here. This means that increasing $\sigma_{\text{brain}}$ within the range of values we are exploring does not degrade our spatial resolution dramatically.

The selection of $\alpha_{\text{spatial}}$ for the subsequent analyses was made by identifying the value of $\alpha_{\text{spatial}}$ that yields the largest AUC. When no spatial bases are used, an $\alpha_{\text{spatial}}$ of $10^{-3}$ gave the highest AUC. In the case where spatial bases are used, the selected value changes depending on the value of $\sigma_{\text{scalp}}$. A value of 1 mm for $\sigma_{\text{scalp}}$ gives the highest AUC across all values of $\alpha_{\text{spatial}}$ and $\sigma_{\text{brain}}$, which suggests that it should yield the best quality image reconstruction. In particular, an $\alpha_{\text{spatial}}$ selection of $10^{-2}$ gave the highest AUC across all values of $\sigma_{\text{brain}}$.

For larger values of $\sigma_{\text{scalp}}$, there is no clear optimal value of $\alpha_{\text{spatial}}$ although there is a general decrease in the AUC as $\alpha_{\text{spatial}}$ increases. In subsequent analysis, we continue to explore all values of $\sigma_{\text{scalp}}$ and $\sigma_{\text{brain}}$ while using an $\alpha_{\text{spatial}}$ of $10^{-2}$ whenever spatial basis functions were used and an $\alpha_{\text{spatial}}$ of $10^{-3}$ when they were not used.

##### Image metrics—single wavelength

**Fig. 3:**
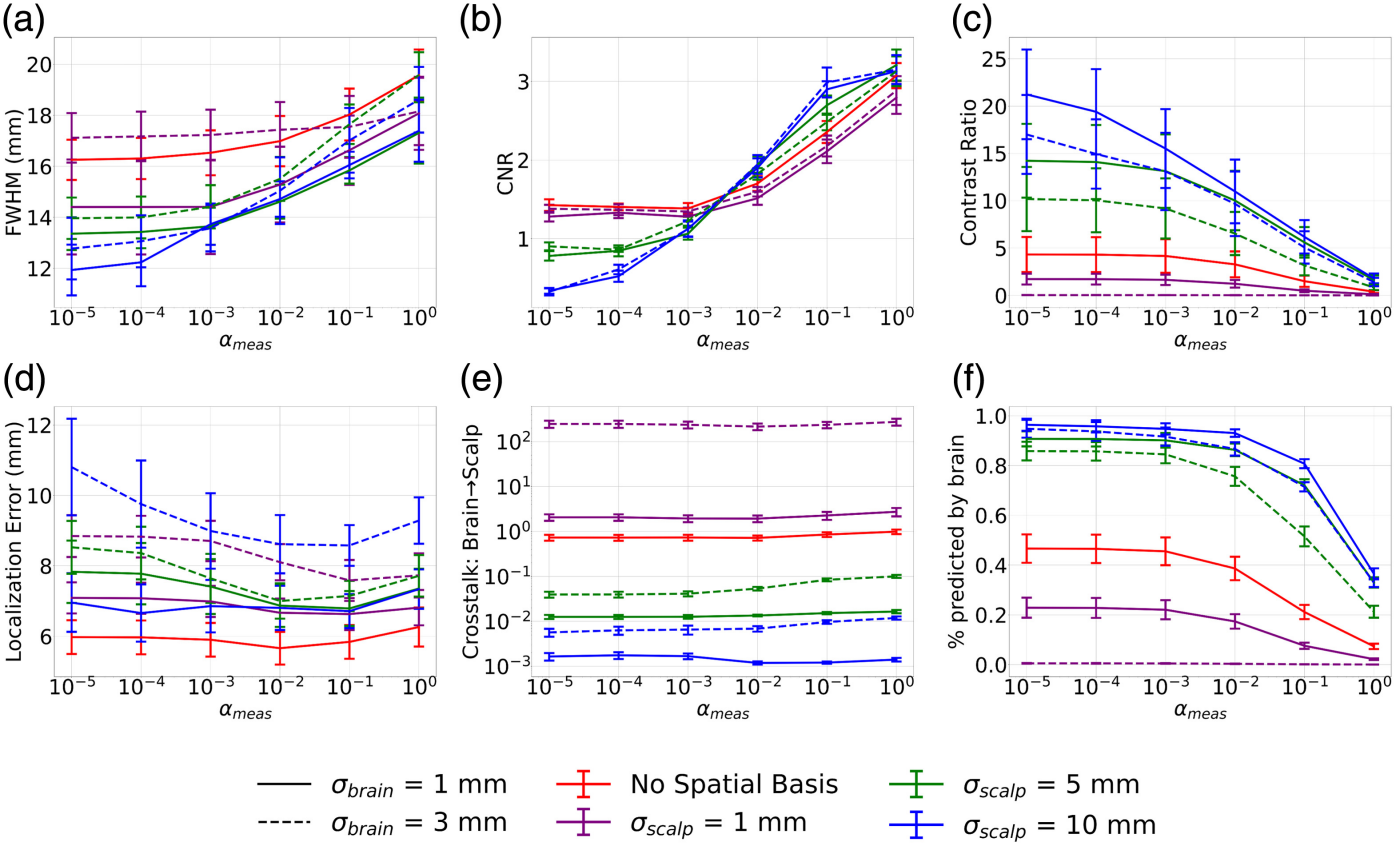
Image quality metrics obtained using a single wavelength (850 nm) for image reconstruction as $\alpha_{\mathrm{meas}}$ is swept from $10^{-5}$ to 1. Activation is modelled using a 3 mm Gaussian blob centered on a given seed vertex on the cortical surface. Varying line style corresponds to varying $\sigma_{\text{brain}}$ and varying color corresponds to varying $\sigma_{\text{scalp}}$. Plots indicate the mean across seed vertices, and error bars correspond to the standard error. (a) Full width half maximum (FWHM) shows an increase as $\alpha_{\mathrm{meas}}$ increases. (b) Contrast-to-noise ratio (CNR) shows an increase as $\alpha_{\mathrm{meas}}$ increases. (c) Contrast ratio decreases as $\alpha_{\mathrm{meas}}$ increases. (d) Localization error increases with increasing $\sigma_{\text{brain}}$. When $\sigma_{\text{brain}}$ is 5 mm, there is an increase in localization error at small and large $\alpha_{\mathrm{meas}}$; otherwise, the localization error is relatively constant. (e) Crosstalk between the brain and scalp is relatively constant across all $\alpha_{\mathrm{meas}}$ but decreases significantly when spatial basis functions are used with $\sigma_{\text{scalp}}$ greater than 1 mm. (f) Percent predicted by the brain decreases when $\alpha_{\mathrm{meas}}$ increases but is higher when spatial basis functions are used with $\sigma_{\text{scalp}}$ greater than 1 mm.

Figure 3 shows the single-wavelength metrics outlined in Table 2 as $\alpha_{\mathrm{meas}}$ is swept from $10^{-5}$ to 1. The ground truth was generated using a 3 mm Gaussian blob of activation. The maximum amplitude of the absorption change in channel space was set such that a maximum change in optical density of 0.02 was measured on the channel with maximum sensitivity to the absorption change. The FWHM of the reconstructed image increased with increasing $\alpha_{\mathrm{meas}}$ because the image is smoothed by the increased measurement regularization [Fig. 3(a)]. FWHM decreased with decreasing $\sigma_{\text{brain}}$ and increasing $\sigma_{\mathrm{scalp}}$, indicating that the selection of a larger $\sigma_{\text{scalp}}$ and smaller $\sigma_{\text{brain}}$ will yield a sharper focus of the target activation. In general, using spatial basis functions yields a smaller FWHM compared with when no spatial basis functions are used, except for the case when $\sigma_{\text{brain}}$ is 5 mm, indicating that at this size, the spatial basis functions on the brain are worsening the spatial resolution of the images.

In general, Fig. 3(b) shows that CNR increases as $\alpha_{\mathrm{meas}}$ increases, but the impact of $\sigma_{\text{brain}}$ and $\sigma_{\text{scalp}}$ on CNR for a given $\alpha_{\mathrm{meas}}$ is variable. For $\alpha_{\mathrm{meas}}$ below $10^{-2}$, spatial bases have a negative impact on CNR relative to when no spatial bases are used, and increasing $\sigma_{\text{scalp}}$ leads to worse CNR. As $\alpha_{\mathrm{meas}}$ increases above $10^{-2}$, we observe the reverse of this trend. The spatial bases improve the CNR particularly for $\sigma_{\text{scalp}}$ greater than 1 mm. Changing $\sigma_{\text{brain}}$ has no noticeable impact on CNR.

The localization error stays relatively constant for all $\alpha_{\mathrm{meas}}$ when no spatial bases are used and for sufficiently small $\sigma_{\text{brain}}$ [Fig. 3(d)]. A selection of $\sigma_{\text{brain}}$ of 3 mm leads to a larger localization error, particularly for smaller $\alpha_{\mathrm{meas}}$. The size of the original blob of activation is 3 mm, so when larger spatial bases are used on the brain, it becomes more difficult to reconstruct the original centroid location of the activation.

Crosstalk between the brain and scalp is significantly impacted using spatial bases. Figure 3(e) shows that when $\sigma_{\text{scalp}}$ becomes greater than 1 mm, crosstalk can be reduced by several orders of magnitude, and the effect becomes more pronounced as $\sigma_{\text{scalp}}$ increases. There is a decrease in crosstalk by ∼3 orders of magnitude between the case where no spatial bases are used and when $\sigma_{\text{scalp}}$ is 10 mm. A reduction in $\sigma_{\text{brain}}$ for a given $\sigma_{\text{scalp}}$ also reduces crosstalk—reducing $\sigma_{\mathrm{brain}}$ from 3 to 1 mm can provide a reduction in crosstalk by roughly 1 order of magnitude. The crosstalk is relatively constant across all values of $\alpha_{\mathrm{meas}}$.

The percentage of the measurement reconstructed on the brain shown in Fig. 3(f) provides an additional measure of the impact of crosstalk between brain and scalp by looking at the channel space projection of the reconstructed brain image. A higher percentage predicted by the brain means that more of the ground truth brain activation had been accurately reconstructed on the brain surface. The trends reflect the same behavior as what was seen in the crosstalk between the brain and scalp. A selection of $\sigma_{\text{scalp}}$ greater than 1 mm increases the percentage reconstructed on the brain compared with when no spatial bases are used, whereas $\sigma_{\text{brain}}$ has a smaller impact. There is a significant drop off in the percentage reconstructed on the brain when $\alpha_{\mathrm{meas}}$ increases past $10^{-2}$. This trend is also reflected in the contrast ratio in Fig. 3(c).

The contrast ratio is a measure of how well the amplitude of the ground truth image is preserved in the reconstructed image [Fig. 3(c)]. This is impacted both by the FWHM—which spatially smooths the reconstructed image and, as a result, reduces contrast—and crosstalk between the brain and scalp—which distributes the original amplitude on the brain between the brain and scalp surfaces. As FWHM increases with an increase in $\alpha_{\mathrm{meas}}$, the contrast ratio decreases. As crosstalk decreases with a decrease in $\sigma_{\text{brain}}$ and increase in $\sigma_{\text{scalp}}$, contrast ratio increases.

It is critical to balance sufficiently high CNR and sufficiently small FWHM while mitigating crosstalk between the brain and scalp when selecting the optimal parameters. The results shown in Fig. 3 indicate that the optimal parameters are $\sigma_{\text{brain}}$ of 1 mm and $\sigma_{\text{scalp}}$ of 5 mm. In the range of $\alpha_{\mathrm{meas}}$ greater than $10^{-2}$, this combination both reduces the FWHM and improves the CNR compared with when no spatial basis functions are used. Furthermore, it reduces the crosstalk between the brain and the scalp by an order of magnitude compared with when no spatial basis functions are used, without causing an increase in the localization error. This combination of $\sigma_{\text{brain}}$ and $\sigma_{\text{scalp}}$ is investigated further in subsequent analyses of multiwavelength image reconstruction and compared with the case when no spatial basis functions are used.

Interestingly, Fig. 2 indicates that a $\sigma_{\text{scalp}}$ of 1 mm would provide better-quality image reconstructions according to the AUC of the singular value spectrum. It is higher in this case compared with all other values of $\sigma_{\text{scalp}}$ and shows a prominent peak at an $\alpha_{\text{spatial}}$ of $10^{-2}$. When evaluating the other metrics of image quality in Fig. 3, a $\sigma_{\text{scalp}}$ of 1 mm does not generally yield better CNR, FWHM, or brain to scalp crosstalk compared with larger values of $\sigma_{\text{scalp}}$. According to Fig. 2, for $\sigma_{\text{scalp}}$ larger than 1 mm, there is no distinct value of $\alpha_{\text{spatial}}$ that will give the highest AUC. This is reflected in Fig. S1 in the Supplementary Material, which shows that $\alpha_{\text{spatial}}$ does not significantly affect the CNR of the images. Figures S1(c) and S1(e) in the Supplementary Material show that a higher $\alpha_{\text{spatial}}$ will reduce contrast ratio and increase crosstalk between the brain and scalp, respectively. This is because smaller $\alpha_{\text{spatial}}$ will push the activation toward deeper, less sensitive vertices in the image reconstruction, which will reduce the amplitude in the scalp and increase the amplitude on the brain and as a result, decrease brain-to-scalp crosstalk, and increase contrast ratio. When $\alpha_{\text{spatial}}$ becomes too small, however, there is an increase in the localization error because the activation will be pushed to deep brain vertices that were not a part of the initial brain activation. This trend is consistent when both spatial basis functions and no spatial basis functions are being used. These results indicate that our selection of $\alpha_{\text{spatial}}$ of $10^{-2}$ for spatial basis functions and $10^{-3}$ when they are not used balances the minimization of localization error and brain-to-scalp crosstalk.

##### Image metrics—dual wavelength

**Fig. 4:**
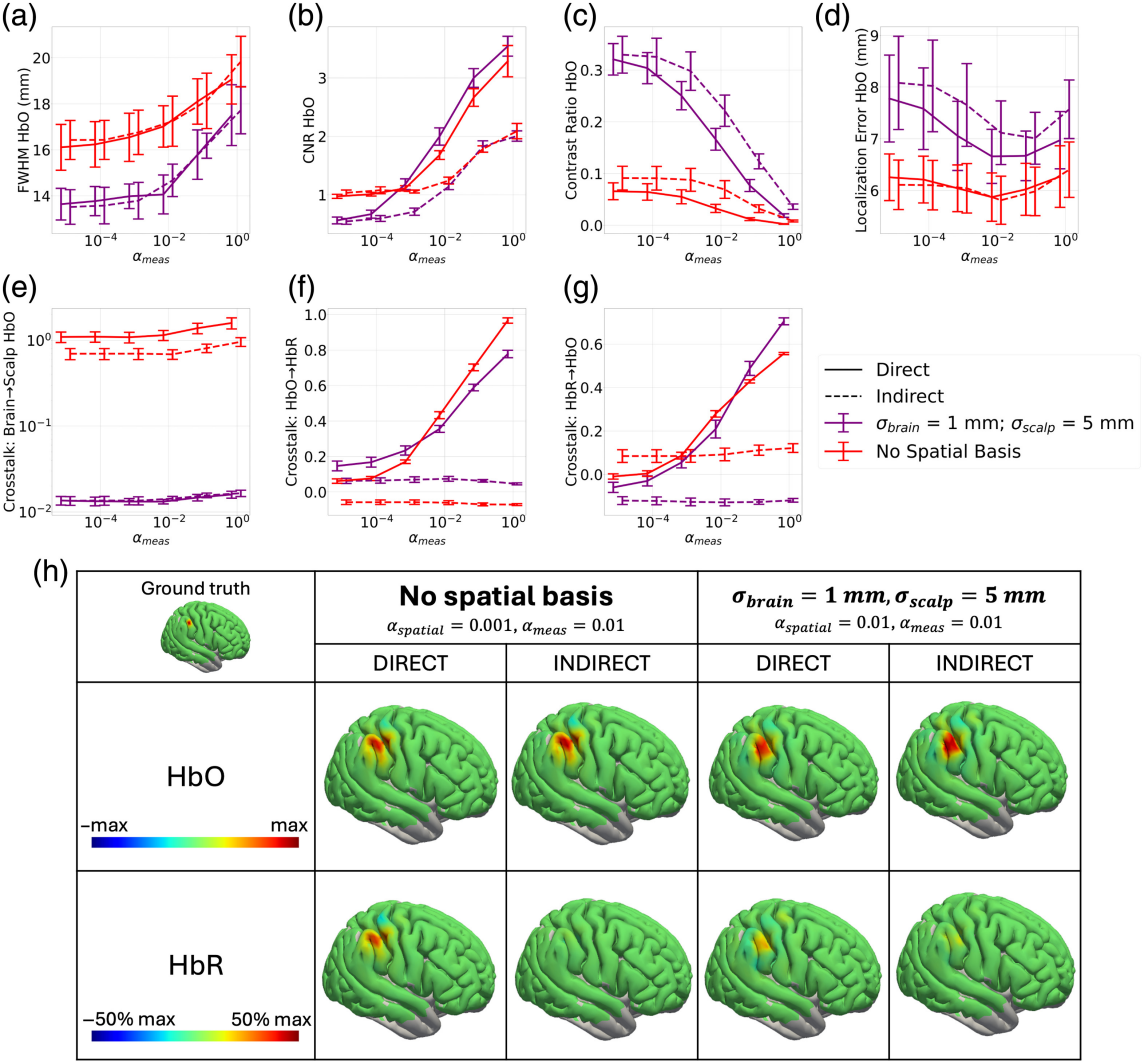
(a)–(g) Image quality metrics obtained using the activation of a single chromophore when both wavelengths are used to obtain the reconstruction of ΔHbO and ΔHbR as $\alpha_{\mathrm{meas}}$ is swept from $10^{-5}$ to 1. Activation is modelled using a 3 mm Gaussian blob centered on a given seed vertex on the cortical surface. Solid line corresponds to the direct method, whereas dashed line corresponds to the indirect method, and varying color corresponds to varying $\sigma_{\text{scalp}}$. Plots indicate the mean across seed vertices and error bars correspond to the standard error. (a) FWHM of the ΔHbO image. (b) CNR of the ΔHbO image. (c) Contrast ratio of the ΔHbO image. (d) Localization error of the ΔHbO image. (e) Crosstalk between the brain and scalp of the ΔHbO image. (f) Crosstalk of ΔHbO into ΔHbR when ΔHbO is used as the ground truth image. (g) Crosstalk of ΔHbR into ΔHbO when ΔR is used as the ground truth image. (h) Example images for an example $\alpha_{\mathrm{meas}}$. A 3 mm Gaussian blob was modelled as the ground truth image of ΔHbO. The images were reconstructed using varying approaches to illustrate the crosstalk between the image reconstruction of ΔHbO and ΔHbR. For both the case where no spatial basis functions are used and when they are used, there is a higher amplitude in the ΔHbR image for the direct method compared with the indirect method.

Using the selection of $\sigma_{\text{brain}}$ and $\sigma_{\text{scalp}}$ made above, the same metrics were evaluated when using both wavelengths to reconstruct ΔHbO and ΔHbR. This allowed us to evaluate the impact of using the direct and indirect methods outlined in Sec. 2.1 on the metrics of image quality using the image of ΔHbO when it was used as the ground truth activation. We were further able to evaluate the impact of the two methods on the crosstalk between the chromophores using both ΔHbO and ΔHbR as the ground truth activations. Figure 4(f) indicates that the indirect method leads to less crosstalk of ΔHbO into ΔHbR both with and without spatial bases. This holds true across all values of $\alpha_{\mathrm{meas}}$. The amount of crosstalk for the direct method increases as $\alpha_{\mathrm{meas}}$ increases, whereas for the indirect method, it remains relatively constant. This indicates that, especially when using larger $\alpha_{\mathrm{meas}}$, there is a significant advantage to using the indirect method to mitigate chromophore crosstalk. This is highlighted in Fig. 4(h), which shows example images of ΔHbO and ΔHbR to demonstrate the significance of the crosstalk on the image reconstructions. The trend is similar when looking at the crosstalk of ΔHbR into ΔHbO, but overall, the amount of crosstalk is less [Fig. 4(g)].

That said, there appears to be a cost in terms of CNR [Fig. 4(b)] when the indirect method is used. The CNR is higher for the direct method than the indirect method at $\alpha_{\mathrm{meas}}$ greater than $10^{-2}$. All other metrics—FWHM, localization error, contrast ratio, and brain to scalp crosstalk—show similar trends to those in Fig. 3. In these metrics, the dominant factor influencing image reconstruction quality was the use of spatial basis functions and not the choice between the direct or indirect reconstruction method.

#### 3.1.2 Parameter selection validation using an augmented dataset

##### Image metrics—single wavelength

**Fig. 5:**
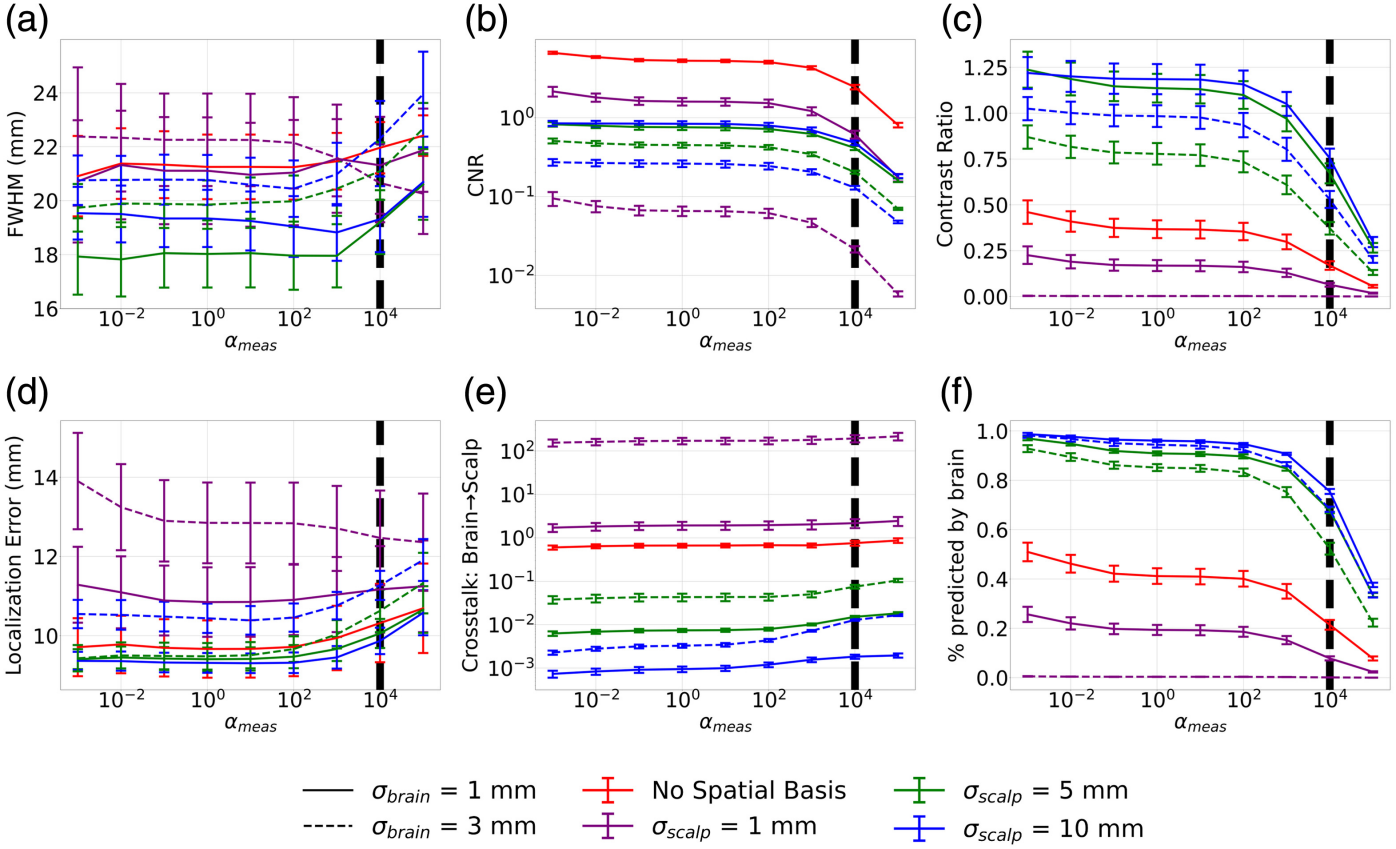
Metrics obtained using a single wavelength (850 nm) for image reconstruction as $\alpha_{\mathrm{meas}}$ is swept from $10^{-3}$ to $10^{5}$ for the augmented resting state dataset. Activation is modelled using a 15 mm Gaussian blob centered on a given seed vertex on the cortical surface. Varying line style corresponds to varying $\sigma_{\text{brain}}$ and varying color corresponds to varying $\sigma_{\text{scalp}}$. Plots indicate the mean across seed vertices, and error bars correspond to the standard error. The dashed black line indicates the value of $\alpha_{\mathrm{meas}}$, which yields a value of $\lambda_{\mathrm{meas}}=1$ given $\lambda_{R}=10^{-6}$. (a) Full width half maximum (FWHM) roughly increases as $\alpha_{\mathrm{meas}}$ increases. (b) Contrast-to-noise ratio (CNR) shows a decrease as $\alpha_{\mathrm{meas}}$ increases. Using no spatial basis functions yields the highest CNR. (c) Contrast ratio decreases as $\alpha_{\mathrm{meas}}$ increases. Using spatial basis functions yields a higher contrast ratio. (d) Localization error increases as $\alpha_{\mathrm{meas}}$ increases. (e) Crosstalk between the brain and scalp is relatively constant across all $\alpha_{\mathrm{meas}}$ but decreases significantly when spatial basis functions are used with $\sigma_{\text{scalp}}$ greater than 1 mm. (f) Percent predicted by the brain decreases when $\alpha_{\mathrm{meas}}$ increases but is higher when spatial basis functions are used with $\sigma_{\text{scalp}}$ greater than 1 mm.

To properly evaluate the quality of the image reconstruction under experimental noise conditions, augmented resting state data were used. We increased the standard deviation of the Gaussian blob of activation from 3 mm to 15 mm in this section of the simulation study to more accurately reflect the size of a true detectable brain activation and to produce a larger signal change across more channels to better handle variability in noise across channels. The range of $\alpha_{\mathrm{meas}}$ investigated here shifted toward higher values as a result of incorporating measurement covariances in Eq. (11). These covariance values are typically on the order of $10^{-6}$, so larger values of $\alpha_{\mathrm{meas}}$ can be used to achieve measurement regularization on a similar scale to when the identity matrix is being used. The dashed black line in Fig. 5 indicates the value of $\alpha_{\mathrm{meas}}$ that yields a value of $\lambda_{\mathrm{meas}}$ of 1. As mentioned in Sec. 2.5.4, this is the value of $\alpha_{\mathrm{meas}}$ where we can have full confidence in our estimate of the posterior covariance and, therefore, confidence in the CNR.

The metrics in Fig. 5 have similar trends to those in Fig. 3. Spatial basis functions, when $\sigma_{\text{scalp}}$ was greater than 1 mm, improve the crosstalk between the brain and the scalp significantly [Fig. 5(e)]. This is also reflected in the percentage predicted by the brain [Fig. 5(f)]. Using spatial basis functions with $\sigma_{\text{scalp}}$ greater than 1 mm results in more activation being predicted by the brain, but there is a reduction in this percentage as $\alpha_{\mathrm{meas}}$ increases. This is correspondingly reflected in the reduction in contrast ratio seen as $\alpha_{\mathrm{meas}}$ increases, $\sigma_{\text{brain}}$ increases, and $\sigma_{\text{scalp}}$ decreases [Fig. 5(c)].

Localization error remains relatively constant, with a slight increase as $\alpha_{\mathrm{meas}}$ increases [Fig. 5(d)]. Having $\sigma_{\text{scalp}}$ at 1 mm or $\sigma_{\text{brain}}$ at 5 mm results in a much higher localization error. FWHM increases slightly as $\alpha_{\mathrm{meas}}$ increases as expected. There is also a small increase in FWHM as $\sigma_{\text{brain}}$ increases [Fig. 5(a)].

The CNR obtained from the augmented resting state data diverges the most significantly from the results of the simulation. This can be attributed to the difference in the noise modelling used in the simulation (which used white noise) compared with the augmented dataset. Here, we were able to model the noise using real measurement noise that varies considerably across channels because of the effects of scalp coupling as well as spatial variations in the optical properties of the head. That said, when using the posterior covariance as an estimate of the image space within-subject variance, there is a balance between the effect of the image covariance and the measurement covariance. The posterior covariance will always be smaller than the prior, R [Eq. (28)] because there is additional information being incorporated from the data, $C_{\mathrm{meas}}$. As $\alpha_{\mathrm{meas}}$ becomes smaller, we are implying that we have greater trust in the measurements; this leads to a smaller posterior variance and higher CNR. There is a plateau in CNR across sufficiently small values of $\alpha_{\mathrm{meas}}$ because within this range, the posterior covariance approaches zero, and the only contribution to the total noise estimate in image space comes from the between-subject variance. As $\alpha_{\mathrm{meas}}$ becomes larger, the posterior covariance will increase, and there will be a reduction in CNR as both the within-subject and between-subject variances contribute to the total noise estimate. That said, as mentioned in Sec. 2.5.4, when using the Bayesian framework, $C_{\mathrm{meas}}$ is directly interpreted as the variance of the measurements; there should be no scaling using $\lambda_{\mathrm{meas}}$ because $C_{\mathrm{meas}}$ is coming from the data. The estimate of the posterior variance is therefore only valid at $\alpha_{\mathrm{meas}}$ of $10^{4}$, indicated using the dashed black line.

Using this value of $\alpha_{\mathrm{meas}}$ as reference, conclusions can still be drawn on the effect of spatial basis functions on CNR. Unlike Fig. 3(b), the CNR is highest when no spatial basis functions are used across all values of $\alpha_{\mathrm{meas}}$, and it decreases as $\sigma_{\text{brain}}$ increases and $\sigma_{\text{scalp}}$ decreases.

As mentioned above, Fig. 2 indicates that a $\sigma_{\text{scalp}}$ of 1 mm should give the best-quality image reconstruction according to the metric of AUC. However, similar to Fig. 3, Fig. 5 indicates that a $\sigma_{\text{scalp}}$ of 1 mm does not yield the best CNR, FWHM, or brain to scalp crosstalk compared with larger values of $\sigma_{\text{scalp}}$. Figure S2 in the Supplementary Material shows the effect of $\alpha_{\text{spatial}}$ on the image quality metrics for the augmented resting state data and, as in Fig. S1 in the Supplementary Material, the results indicate that our selection of $\alpha_{\text{spatial}}$ of $10^{-2}$ for spatial basis functions and $10^{-3}$ when they are not used balances the minimization of localization error and brain-to-scalp crosstalk.

These results point to an optimal combination of $\sigma_{\text{brain}}$ of 1 mm and $\sigma_{\text{scalp}}$ of 5 mm if the goal is to minimize crosstalk between the brain and scalp while maintaining sufficiently high CNR and sufficiently small FWHM. It is evident, however, that there will be a penalty for CNR when spatial basis functions are used. Both this optimal set of $\sigma_{\text{brain}}$ and $\sigma_{\text{scalp}}$ and no spatial basis functions are investigated further below.

##### Image metrics—dual wavelength

**Fig. 6:**
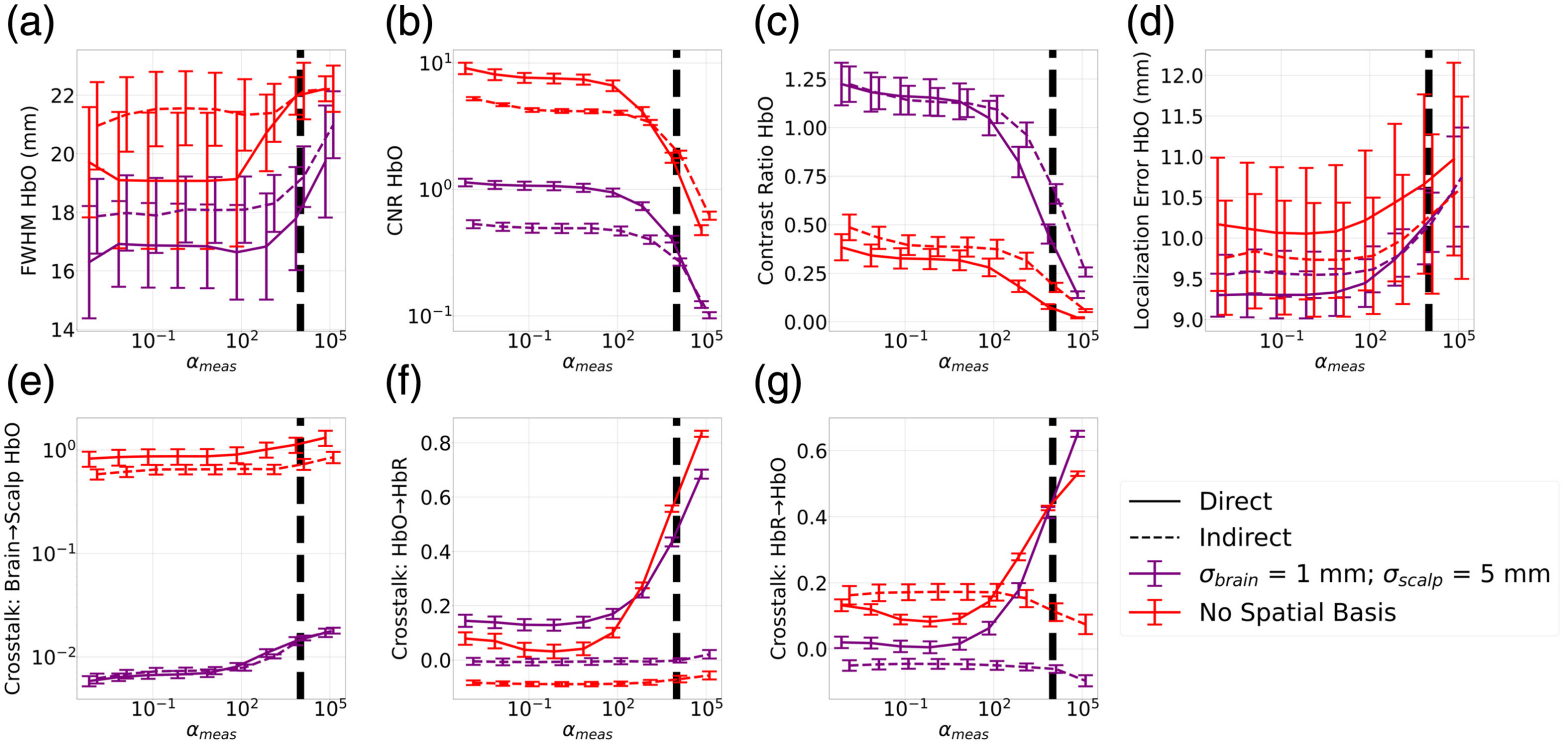
Image quality metrics obtained using the activation of a single chromophore when both wavelengths are used to obtain the reconstruction of ΔHbO and ΔHbR as $\alpha_{\mathrm{meas}}$ is swept from $10^{-3}$ to $10^{5}$ for the augmented resting state dataset. Activation is modelled using a 15 mm Gaussian blob centered on a given seed vertex on the cortical surface. Solid line corresponds to the direct method, whereas dashed line corresponds to the indirect method, and varying color corresponds to varying $\sigma_{\text{scalp}}$. Plots indicate the mean across seed vertices, and error bars correspond to the standard error. The dashed black line indicates the value of $\alpha_{\mathrm{meas}}$, which yields a value of $\lambda_{\mathrm{meas}}=1$ given $\lambda_{R}=10^{-6}$. (a) FWHM of the ΔHbO image. (b) CNR of the ΔHbO image. (c) Contrast ratio of the ΔHbO image. (d) Localization error of the ΔHbO image. (e) Crosstalk between the brain and scalp of the ΔHbO image. (f) Crosstalk of ΔHbO into ΔHbR when ΔHbO is used as the ground truth image. (g) Crosstalk of ΔHbR into ΔHbO when ΔHbR is used as the ground truth image.

Figure 6 is analogous to Figs. 4(a)–4(g) but using the images from the augmented resting state dataset. It again shows the impacts of the direct and indirect methods on the image reconstruction quality for both when no spatial basis functions and when the optimal set of the spatial basis function parameters are used.

The metrics reflect similar trends to Figs. 4(a)–4(g) for most metrics except, predictably, CNR. Figure 4(b) indicates that the direct method resulted in improved CNR over the indirect method for $\alpha_{\mathrm{meas}}$ greater than $10^{-2}$, whereas the use of spatial basis functions had little impact. Here, in Fig. 6(b), both the direct and indirect methods result in better CNR when no spatial basis functions are used compared with when they are used. This means that for CNR, FWHM, localization error, contrast ratio, and crosstalk of the brain into the scalp, the dominant factor in determining the image reconstruction quality is the use of spatial basis functions, not the choice of the indirect or direct method. Spatial basis functions improve the crosstalk between the brain and scalp and the contrast ratio at the expense of worse localization error and CNR.

The crosstalk between ΔHbO and ΔHbR is still an important consideration, and here, we do see the impact of using the direct and indirect methods. When $\alpha_{\mathrm{meas}}$ becomes greater than $10^{2}$, there is a significant increase in the amount of crosstalk for the direct method, whereas the indirect method remains relatively constant [Fig. 5(f)]. Furthermore, to trust the CNR, a value of $\alpha_{\mathrm{meas}}$ of $10^{4}$ must be used. With this constraint on the selection of $\alpha_{\mathrm{meas}}$, the indirect method will yield better results in terms of ΔHbO and ΔHbR  crosstalk without sacrificing image quality in regard to the other metrics.

By synthesizing all the results above, the optimal image reconstruction requires balancing trade-offs among CNR, chromophore crosstalk, and brain-to-scalp crosstalk. To obtain the best CNR and chromophore crosstalk, the indirect method with $\alpha_{\mathrm{meas}}$ of $10^{4}$, no spatial basis, and $\alpha_{\text{spatial}}$ of $10^{-3}$ should be used. However, to minimize brain-to-scalp crosstalk, it is best to use spatial basis functions with a $\sigma_{\text{brain}}$ of 1 mm a $\sigma_{\text{scalp}}$ of 5 mm and an $\alpha_{\text{spatial}}$ of $10^{-2}$. We continued to evaluate the four parameter combinations used in Figs. 4 and 6 to validate the simulation studies on real data and to continue to compare the effect of spatial basis functions and the direct versus indirect methods.

### 3.2 Ball-Squeezing Task

**Fig. 7:**
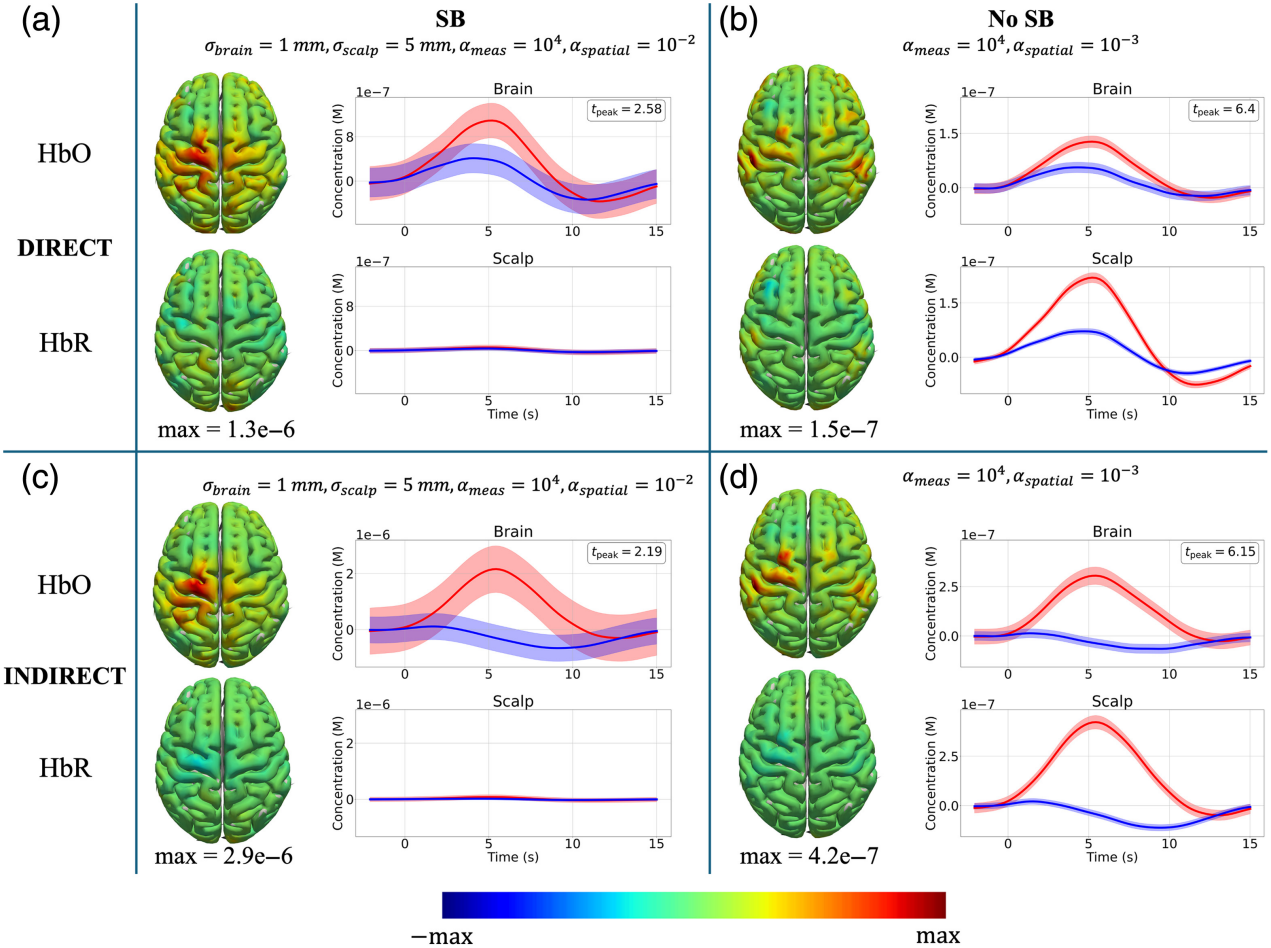
Within each quadrant, the top image contains the magnitude images of the group average for ΔHbO for the right-handed ball-squeezing task. The bottom row contains the magnitude images of the group average. The timeseries plot shows the group average HRF timeseries within the ROI containing vertices with magnitude greater than 50% of the max vertex magnitude in the group-averaged ΔHbO image. The timeseries is shown for ΔHbO (red) and ΔHbR (blue) for the brain (top) and the scalp (bottom). The shaded region corresponds to the total standard error (both within and between subjects). The value of $t_{\text{peak}}$ denoted in the top right corner corresponds to the t-statistic calculated after taking the mean of the group-averaged timeseries and the total standard error over the window from 5 to 8 s for the ΔHbO image. The t-statistic corresponding to p=0.05 for n=17 is 2.12. (a) Direct method, with spatial basis functions. (b) Direct method, no spatial basis functions. (c) Indirect method, with spatial basis functions. (d) Indirect method, no spatial basis functions.

The ball-squeezing task was designed to evoke a strong, bilateral motor response in all subjects to validate the results shown in the simulation study. Figure 7 shows images of the magnitude of the group-averaged results from the right-handed ball squeeze task (left-handed results can be seen in Fig. S3 in the Supplementary Material) across four different parameter combinations. The timeseries of the brain and scalp HRF are obtained from the ROI containing vertices in the brain and scalp magnitude images with amplitude greater than 50% of the max vertex amplitude. The value of $t_{\text{peak}}$ denoted in the top right corner of the timeseries plots indicates the t-statistic of the HRF timeseries when both the mean and standard error are averaged over the window of 5 to 8 s, corresponding to the peak of the HRF.

Overall, the images of the magnitude show localized activation in the contralateral (left) motor and pre-motor cortices corresponding to an HRF timeseries that shows the expected temporal dynamics for a 5 s task. The activation is well localized in all four parameter combinations. When no spatial basis functions are used, it is also possible to identify activation in the ipsilateral (right) hemisphere. There are some deviations in the activation from the exact motor cortex locations, but this can be attributed to individual variations in head shape and cap placement.

The trends seen between the different sets of parameters in the simulation studies are reflected in this validation dataset. First, we will compare the image reconstruction when spatial bases are used compared with when they are not used (Fig. 7, left versus right). The use of spatial bases reduces the crosstalk between the brain and scalp. This is evident in the HRF timeseries and the maximum amplitude in the magnitude images. When spatial basis functions are used, the HRF reconstructed on the scalp has an amplitude near zero. This allows nearly all the amplitude of the HRF to be reconstructed in the brain. Conversely, when no spatial basis functions are used, the magnitude of the activation becomes split between the brain and scalp surfaces. This reduces the magnitude of the activation in the brain by approximately an order of magnitude. This confirms the trends seen in Figs. 3–6, which indicated that using spatial basis functions would greatly reduce the crosstalk between the brain and scalp.

Figure S4 in the Supplementary Material provides further evidence by presenting images of the ΔHbO magnitude on the scalp surfaces. The percentage of the brain amplitude is calculated as the maximum amplitude of the image of the scalp divided by the maximum amplitude of the image of the brain. Taking the indirect method as an example, the percentage of brain amplitude is 2.8% versus 140% when spatial basis functions are used and not used, respectively. This reinforces the notion that the spatial basis functions allow for more of the HRF magnitude to be reconstructed in the brain.

We do see, however, that despite all conditions having a significant maximum t-statistic ($t_{\text{peak}}>2.12$; p<0.05), the t-statistics are higher when no spatial basis functions are used. This again confirms the trends seen in Figs. 5 and 6, which indicated that a higher CNR could be achieved when no spatial basis functions were used. This also validates the use of using the augmented resting state simulation for the selection of the image reconstruction parameters because the trend for CNR seen in this validation dataset is better reflected in the augmented resting state dataset metrics (Figs. 5 and 6) instead of the simulation using a white noise model (Figs. 3 and 4) due to the more accurate noise modelling.

Next, comparing the direct and indirect methods (Fig. 6, top versus bottom) provides insight into the effect of crosstalk between ΔHbO and ΔHbR. The crosstalk between chromophores is evident in the timeseries for the direct method. The HRF for ΔHbR shows no negativity that is typically associated with an increase in ΔHbO. This is congruent with the augmented resting state simulation results shown in Fig. 5. There is a small trade-off seen here where the direct method yields a higher maximum t-statistic in the ΔHbO timeseries.

## 4 Discussion

DOT has been used for over two decades to map changes in measured optical density to changes in oxy- and deoxygenated hemoglobin concentration in the brain.^51^ This paper establishes a framework for optimizing the image reconstruction pipeline using surface representations of the brain and the scalp—emphasizing the importance of using augmented resting state data for this process to accurately account for the noise structure present in a dataset. By first using augmented resting-state simulations and subsequently a simple motor task, we have been able to validate the pipeline so that it can be extended to more complex experimental designs. This will ultimately lead to the generation of better quality images and allow researchers to gain greater neuroscientific insights. We show this for our hexagonal WH, HD array, but the principles outlined here can be extended to any HD probe. Importantly, this study highlights the effect of using spatial basis functions on image reconstruction quality and the effect of using the direct or indirect method on chromophore crosstalk.

For our dataset, we have landed on the following set of image reconstruction parameters for optimal image quality—using the indirect method with an $\alpha_{\text{spatial}}$ of $10^{-3}$, an $\alpha_{\mathrm{meas}}$ of $10^{4}$, and no spatial basis functions. The justification for this selection will be discussed in the sections below.

### 4.1 Direct Versus Indirect Method

The work in Ref. 31 previously evaluated the impact of the direct and indirect methods on chromophore crosstalk using a transmission geometry. Using a pure simulation, they found that the direct method provided better CNR than the indirect method. This is consistent with Fig. 4(b), which shows that the direct method provides better CNR than the indirect method at values of $\alpha_{\mathrm{meas}}$ greater than $10^{-3}$ and with Fig. 6(b), which shows the same trend when $\alpha_{\mathrm{meas}}$ is less than $10^{4}$.

The work of Li et al.^31^ also found that the direct method reduces chromophore crosstalk compared with the indirect method. In this study, we find the opposite effect for both the simulation and the augmented resting state data. We speculate that this is related to differences in our reflection geometry compared with the transmission geometry used in the earlier work. During image reconstruction, the point spread function spreads spatially across the vertices in the brain and the scalp. For the indirect method, as each wavelength is being reconstructed independently, the spread is contained to only this spatial dimension. For the direct method, however, both chromophores are being reconstructed simultaneously. As a result, there is additional spread of the point spread function across chromophores, which contributes to a higher amount of chromophore crosstalk. Particularly, as $\alpha_{\mathrm{meas}}$ increases, and the image becomes smoother across space, there is also the potential for there to be more smoothing across chromophores, leading to the increase in crosstalk seen for the direct method at higher $\alpha_{\mathrm{meas}}$.

As we chose a value of $\alpha_{\mathrm{meas}}$ of $10^{4}$ across all methods, there is significant crosstalk seen in Fig. 7 when the direct method is used. This causes the ΔHbR timeseries to become positive compared with the indirect method, which shows the expected deactivation. The deactivation of ΔHbR can be overpowered due to the crosstalk from ΔHbO into the ΔHbR image reconstruction when using the direct method. The trade-off in CNR between the two methods was minimal at a value of $\alpha_{\mathrm{meas}}$ of $10^{4}$. These results provide strong evidence to support the use of the indirect method.

### 4.2 Spatial Basis Functions

Spatial basis functions were first introduced in Ref. 32 to both spatially smooth the image reconstruction and to reduce the degrees of freedom of the model, making it more computationally tractable. In this prior work, they used overlapping, 3D Gaussian kernels with a standard deviation of 6 mm to do volumetric image reconstructions limited to only the cortical volume. This was extended in Ref. 19 to do surface reconstructions using overlapping 2D Gaussian kernels with a standard deviation of 5 mm for the brain and 20 mm for the scalp. Here, we wanted to rigorously evaluate the effect of using these spatial basis functions to establish the optimal size of the spatial bases for both the brain and the scalp surfaces.

In general, the strongest effect of using spatial basis functions was the dramatic reduction in crosstalk between the brain and the scalp when $\sigma_{\text{scalp}}$ is ≥5  mm. These larger spatial basis functions for the scalp suppress the potential for brain activity being modeled as a mixture of signals from the brain and scalp and thus result in a more accurate reconstruction of the true brain activity amplitude. This does come, in certain instances, at the expense of CNR. For the pure simulation, when $\alpha_{\mathrm{meas}}$ is below $10^{-2}$, using spatial basis functions worsens the CNR. In this low range of $\alpha_{\mathrm{meas}}$, the noise in the images is not being strongly suppressed. Without spatial basis functions, this noise can be reconstructed in the scalp, leaving the brain images less noisy and boosting CNR. When spatial basis functions are used on the scalp, they do not allow for the reconstruction of noise with a high spatial frequency pattern. This means the noise gets pushed to the brain, worsening the CNR. When $\alpha_{\mathrm{meas}}$ becomes sufficiently large, the image noise is suppressed enough in general. As a result, there is less noise in the brain images, and the factor dictating the CNR becomes the size of the spatial basis functions in the brain. As $\sigma_{\text{brain}}$ becomes smaller, the spatial basis functions are better able to model the expected activation on the brain surface.

In the augmented data, using spatial basis functions always leads to worse CNR compared with when no spatial basis functions are used. We attribute this to the effect of model mismatch, which occurs when the model’s sensitivity to the brain, obtained using the head atlas, does not match the experimental sensitivity to the brain. This systematic error caused by the model inconsistency makes it difficult to model the resting state fluctuations across channels with the current head atlas. When spatial basis functions are not used, these systematic errors can be reconstructed on the scalp. When using spatial basis functions on the scalp, however, these systemic errors affect the reconstruction of the brain, which in turn worsens the CNR. The handling of these systematic errors varies from the random noise seen in the pure simulation. In that case, using a large $\alpha_{\mathrm{meas}}$ was enough to suppress the random noise, allowing the spatial basis functions to improve CNR as mentioned above. The degree to which this phenomenon of model mismatch occurs could depend on the head model used and could be investigated further in future work.

It is worth noting that this is partly a limitation of using surface reconstructions. In the case of the augmented resting state data, the model is being asked to explain resting state variation in the data using only brain and scalp surfaces. Volumetric image reconstruction does not have this same spatial constraint, which allows for a better fit between the model sensitivity and the experimental sensitivity. Similarly, there is no model mismatch in the simulation because the activation and noise are only simulated on the brain surface, so the simulated data are already constrained to the surfaces prior to reconstruction, leading to less model mismatch.

Figure S4 in the Supplementary Material shows example images of the standard error of ΔHbO on the scalp surface obtained from both the between-subject and within-subject variances in the right-handed ball-squeezing data. This provides insight into the spatial frequencies of the noise that can be reconstructed when spatial basis functions are being used. The standard error is uniformly distributed over the surface of the scalp when spatial basis functions are used, whereas when no spatial basis functions are used, higher-frequency spatial patterns of noise are reconstructed on the scalp. This means that these higher-frequency spatial patterns will not be reconstructed in the brain, boosting the CNR. With spatial basis functions, the inability of the scalp to reconstruct these high-frequency noise components means that they will be reconstructed in the brain, reducing CNR.

This presents an interesting trade-off between brain-to-scalp crosstalk and CNR. To get the benefits of reducing the crosstalk of the target activation between the brain and scalp surfaces, there is an associated drop in CNR as more noise will also be reconstructed on the brain surface. The decision whether to use spatial basis functions will therefore depend on the noise in the data and the magnitude of the brain activation. In the case of the ball-squeezing dataset, we show that while using the indirect method, the spatial basis functions still provide sufficiently high CNR to achieve a significant t-statistic while minimizing the crosstalk between the brain and scalp, reflected in the HRF timeseries (Fig. 7).

### 4.3 Using Measurement Covariance

#### 4.3.1 In the pseudoinverse formulation

The use of a measurement prior to the formulation of the pseudoinverse is well established.^38^ It provides the benefit of scaling the regularization in directions of higher variance so that noisier measurements become more strongly regularized. This also means we no longer need to prune noisy channels prior to processing the data because these channels will be suppressed during the image reconstruction step. This minimizes the bookkeeping required to keep track of the channels pruned per subject and has further benefits in group statistics, which are discussed further below.

It is important to note that the parameter optimization done here was dependent on the noise properties of our dataset. The scaling of $\alpha_{\mathrm{meas}}$ will depend on the average channel variance in a given dataset. As such, it is recommended that the parameters used for the image reconstruction pipeline be re-optimized if the noise properties of a dataset vary significantly from those used here. The noise properties of a dataset are dependent on the fNIRS system design, the geometry and density of the probe as well as the nature of the experimental paradigm. Changes to any of these factors could affect the optimal parameters for the image reconstruction. The code to run the augmented resting-state simulation has been made publicly available so that researchers are able to optimize their pipelines to suit the measurement noise seen in their datasets.

In this study, we restricted the measurement prior to only include the diagonal terms, which represented the variance within a given channel. In theory, the entire covariance matrix could be used to better model the noise structure in the data and further suppress different image modes during the regularization. In future work, we look forward to evaluating the impact of this on the quality of the image reconstruction. This only works, however, when using the formulation of the pseudoinverse in Eq. (11). Using Eq. (10) requires that the measurement covariance matrix, C, be directly inverted. Given the number of epochs used in our task design, this covariance matrix is rank-deficient and, as a result, not reliably invertible. In Eq. (11), C is not inverted directly; it is instead added to the full rank matrix ${\mathrm{AA}}^{T}$ prior to taking the inverse.

#### 4.3.2 In the group average

We further made use of the within-subject measurement variance, defined as the diagonal of the covariance matrix, by propagating it into image space using the Bayesian formulation to obtain the posterior covariance. This gives a measure of the within-subject variance for each vertex on the brain and scalp surfaces. Here, we used it to get a weighted subject average, where each vertex of each subject is inversely weighted by its variance. This acts to suppress the impact of any bad vertices from individual subjects on the group average. It also means we do not have to prune channels, which avoids the issue of having a different number of subjects contributing to different vertices in the group average.

Our iterative approach uses the within-subject variance first, as the weights for the group average. This is then used to estimate the between-subject variance. The final group average is estimated using the inverse of the sum of the between and the within-subject variances as weights. This follows a similar approach to restricted maximum likelihood (ReML), which aims to find the least biased variance estimates using maximum likelihood estimation.^34^^,^^52^ We have simplified the ReML algorithm by manually estimating the between-subject variance from the first iteration of the group average estimate. We expect that we are overestimating our between-subject variance using this approach because our estimate is biased. This will lead to an underestimation of our t-statistics. The t-statistics we obtain in the ball-squeezing data are sufficiently large that we are not concerned with this underestimation, but an implementation of the ReML algorithm could further improve the image statistics.

Beyond ReML, we also believe our estimate of the posterior covariance could be biasing the image t-statistics. The selection of an image prior R requires assumptions to be made on the structure and magnitude of the image covariance. Equation (40) clearly indicates how the magnitude of R can be directly scaled by the parameter $\lambda_{R}$ to then directly scale the magnitude of the posterior covariance without affecting the magnitude of the contrast image [Eq. (38)]. Similarly, the scale of the posterior will not directly affect the weighted averaging done to obtain the group average contrast image, but it will affect the total standard error and subsequently, the t-statistics. To bypass this, we posit that the use of permutation testing could provide a more robust way of estimating these statistics.^53^ This has been well established in the fMRI literature and could be easily implemented for fNIRS analysis. We leave this to future work.

### 4.4 Ball-Squeezing Task

The ball-squeezing data provided the final verification for which set of parameters would yield the best quality image reconstruction. As mentioned earlier, using spatial basis functions minimized the crosstalk between the brain and the scalp while maintaining significant t-statistics. In all cases, the images provided the appropriate resolution to identify activation in the ΔHbO images in the expected regions. These include the motor and pre-motor cortices in the contralateral hemisphere. When no spatial basis functions are used, this localization is more precise, and it is possible to resolve activation in the ipsilateral hemisphere as well. For ΔHbR, the deactivation is most clear only in the case where the indirect method is used due to the mitigation of chromophore crosstalk.

### 4.5 Limitations

A key limitation of fNIRS is that it measures changes in hemoglobin concentrations rather than absolute hemoglobin concentrations. As a result, reconstructed amplitudes are subject to scaling ambiguity arising from the choice of baseline optical properties, slow signal drift, and inter-individual variability in optical pathlengths that arise from anatomical variability and tissue properties. Although baseline correction and drift modeling reduce these effects, they do not fully eliminate them, and absolute amplitude comparisons should therefore be interpreted with caution.

fNIRS measurements are also inherently device dependent. Differences in hardware characteristics, optode geometry, wavelength selection, and system sensitivity influence signal scaling and depth sensitivity, limiting direct quantitative comparisons across instruments. Although relative task-evoked effects and spatial patterns are expected to generalize across systems, reconstruction parameters should be re-optimized when applying the proposed framework to different devices or experimental configurations.

### 4.6 Future Work

Moving from HD to ultra-HD (UHD) has been shown to provide additional benefits to image quality metrics such as CNR, FWHM, and localization error due to the increase in overlapping measurements.^12^^,^^14^ Ultra-HD refers to any probe that has more than two source-detector separations, shorter separations with a minimal separation of ∼<12  mm, and a higher degree of spatial sampling. The HD probe used in this study was collecting data from the first and second nearest neighbor source-detector separations at 19 and 33 mm, respectively. It is possible to collect data in channels with up to the third nearest neighbor source-detector separations at 50 mm. Although incorporating the third nearest neighbor channels does not quite achieve UHD, it will increase the number of overlapping measurements, and we expect to see the image quality increase. Furthermore, as optode packing improves and denser arrays can be more easily designed, we expect the image quality for UHD probes to also show improved CNR, FWHM, etc.

Finally, as mentioned above, it is possible to use the entire measurement covariance matrix in Eq. (11) as the measurement prior. We expect this to further suppress contributions from noisy channels by better modelling the noise structures in the data. Combined with the use of permutation testing to obtain the t-statistics, we expect to obtain more robust and reliable image statistics.

## 5 Conclusions

Using surface representations of the scalp and brain, this study demonstrates how using Gaussian spatial basis functions for image reconstruction effectively reduces crosstalk between brain and scalp signals at the expense of the CNR. In addition, employing the indirect reconstruction method for hemoglobin concentration estimation minimizes the crosstalk between oxy- and deoxyhemoglobin. We further demonstrate that parameter selection is better optimized using resting-state data augmented with synthetic HRFs and provide a principled framework for this process. In parallel, we utilized an alternate approach to channel pruning using our weighted averaging approach to obtain group-level results and statistics.

## Supplementary Material

10.1117/1.NPh.13.2.025001.s01

## Data Availability

All methods developed in this dataset are available in Cedalion for adoption by the community (https://github.com/ibs-lab/cedalion. All data are available on OpenNeuro (doi:10.18112/openneuro.ds006673.v1.0.2). Code for running the augmented simulation and generating Figs. 5–7 is publicly available on GitHub (https://github.com/lauracarlton/image_reconstruction_optimization.git).
