# Supplementary material for: Surface-based image reconstruction optimization for high-density functional near-infrared spectroscopy
Source: Neurophotonics. 2026 Mar 14;13(2):025001. doi: 10.1117/1.NPh.13.2.025001 (PMC12990250; doi:10.1117/1.NPh.13.2.025001)
Supplement: Supplementary file 1 [file NPh_013_025001_SD001.pdf]

Supplemental Material

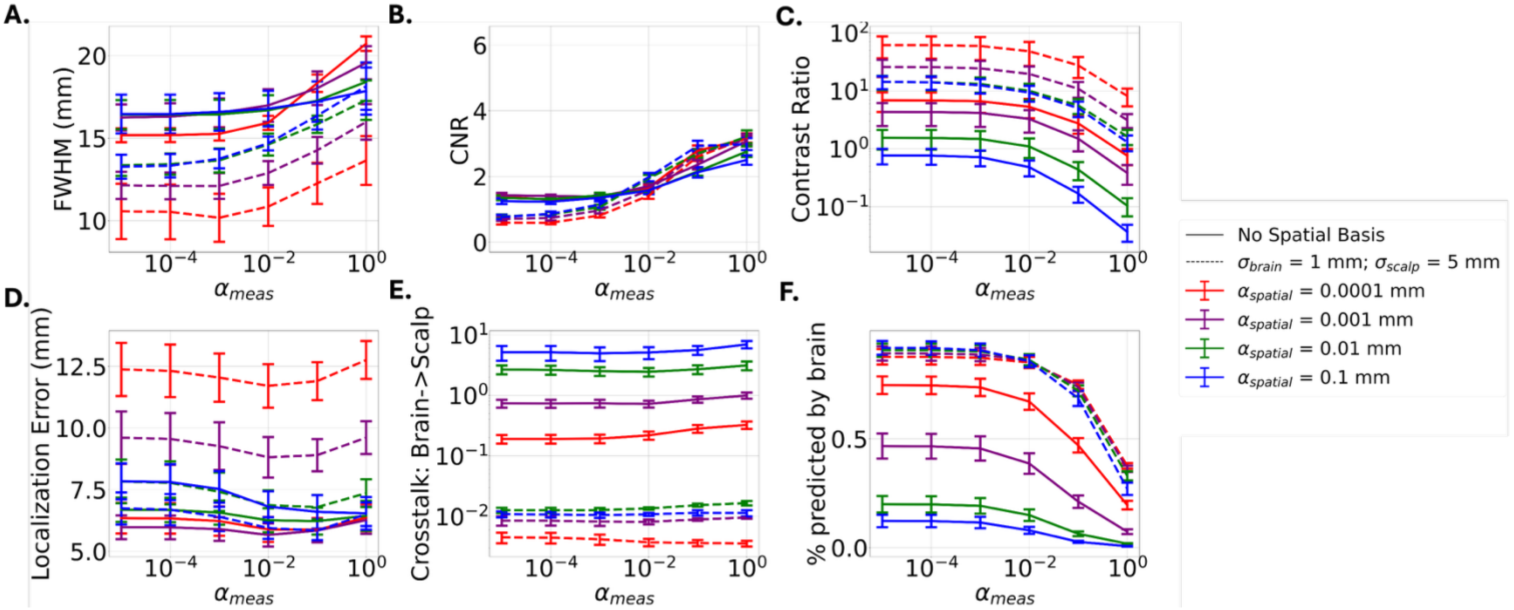

Supp Figure 1. Simulation results using a Gaussian blob of activation with standard deviation of 3 mm as the ground truth. ***A)*** *FWHM. When spatial basis functions are used, the FWHM increases as $\alpha_{spatial}$ increases. When spatial basis functions are not used, there is no strong effect of $\alpha_{spatial}$ on the FWHM.* ***B)*** *CNR. There is no strong effect of $\alpha_{spatial}$ on the CNR.* ***C)*** *Contrast ratio. Both when spatial basis functions are used and not, contrast ratio decreases as $\alpha_{spatial}$ increases.* ***D)*** *Localization error. When spatial basis functions are used, localization error increases as $\alpha_{spatial}$ decreases. Conversely, when spatial basis functions are not used, localization error decreases as $\alpha_{spatial}$ decreases.* ***E)*** *Crosstalk from the brain into the scalp. As $\alpha_{spatial}$ decreases, crosstalk decreases.* ***F)*** *Percent predicted by the brain. When spatial basis functions are used there is no strong effect of $\alpha_{spatial}$ on the percent predicted by the brain. When no spatial basis functions are used, there is a decrease in the percent predicted by the brain as $\alpha_{spatial}$ increases.*

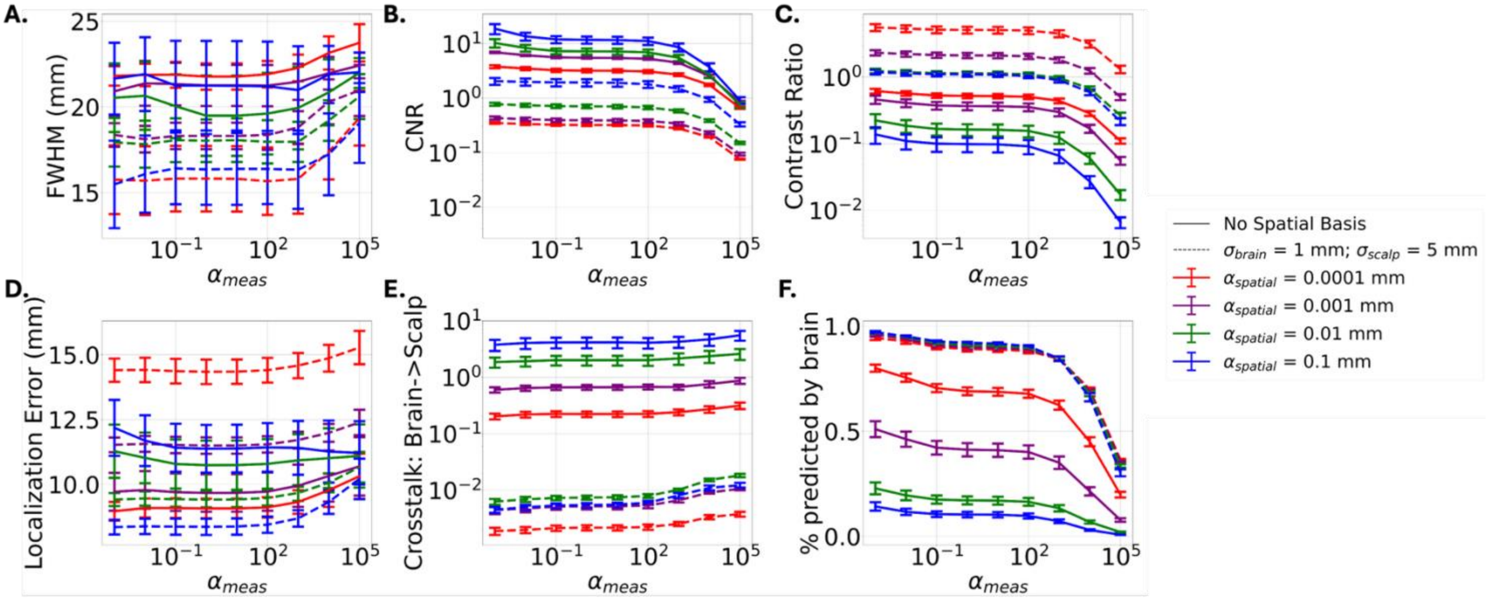

Supp Figure 2. Augmented resting state results using a Gaussian blob of activation with standard deviation of 15 mm as the ground truth. ***A)*** *FWHM. When spatial basis functions are used, there is no trend between $\alpha_{spatial}$ and FWHM. Both the smallest and largest values of $\alpha_{spatial}$ give the smallest FWHM. When spatial basis functions are not used, there is no strong effect of $\alpha_{spatial}$ on the FWHM.* ***B)*** *CNR. There is a small reduction in CNR as $\alpha_{spatial}$ decreseas.* ***C)*** *Contrast ratio. Both when spatial basis functions are used and not, contrast ratio decreases as $\alpha_{spatial}$ increases.* ***D)*** *Localization error. When spatial basis functions are used, localization error increases as $\alpha_{spatial}$ decreases. Conversely, when spatial basis functions are not used, localization error decreases as $\alpha_{spatial}$ decreases.* ***E)*** *Crosstalk from the brain into the scalp. As $\alpha_{spaital}$ decreases, crosstalk decreases.* ***F)*** *Percent predicted by the brain. When spatial basis functions are used there is no strong effect of $\alpha_{spatial}$ on the percent predicted by the brain. When no spatial basis functions are used, there is a decrease in the percent predicted by the brain as $\alpha_{spatial}$ increases.*

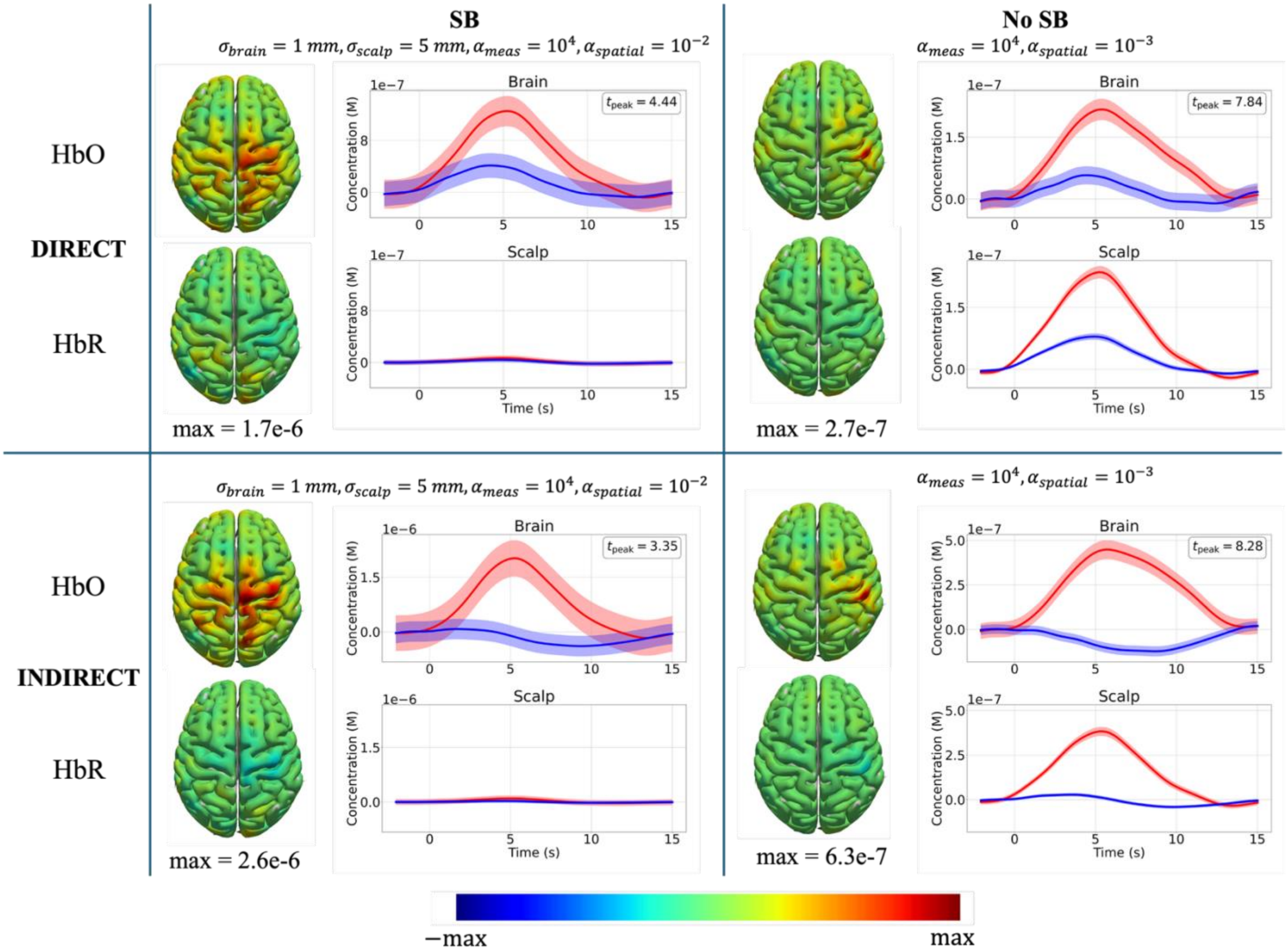

Supp Figure 3. Within each quadrant, the top image contains the magnitude images of the group average for ΔHbO for the right handed ball squeezing task. The bottom row contains the magnitude images of the group average for ΔHbR. The timeseries plot shows the group average HRF timeseries within the ROI containing vertices with magnitude greater than 50% of the max vertex magnitude in the group averaged ΔHbO image. The timeseries is shown for ΔHbO (red) and ΔHbR (blue) for the brain (top) and the scalp (bottom). The shaded region corresponds to the total standard error (both within and between subjects). The value of t_{peak} denoted in the top right corner corresponds to the t-statistic calculated after taking the mean of the group averaged timeseries and the total standard error over the window from 5 to 8 seconds. ***Top Left:*** *Direct method, with spatial basis functions.* ***Top Right:*** *Direct method, no spatial basis functions.* ***Bottom Left:*** *Indirect method, with spatial basis functions.* ***Bottom Right:*** *Indirect method, no spatial basis functions. In general, the responses are smaller than those seen in the right-handed ball squeezing task and the t-statistics are not as high. Otherwise the trends between the methods are consistent.*

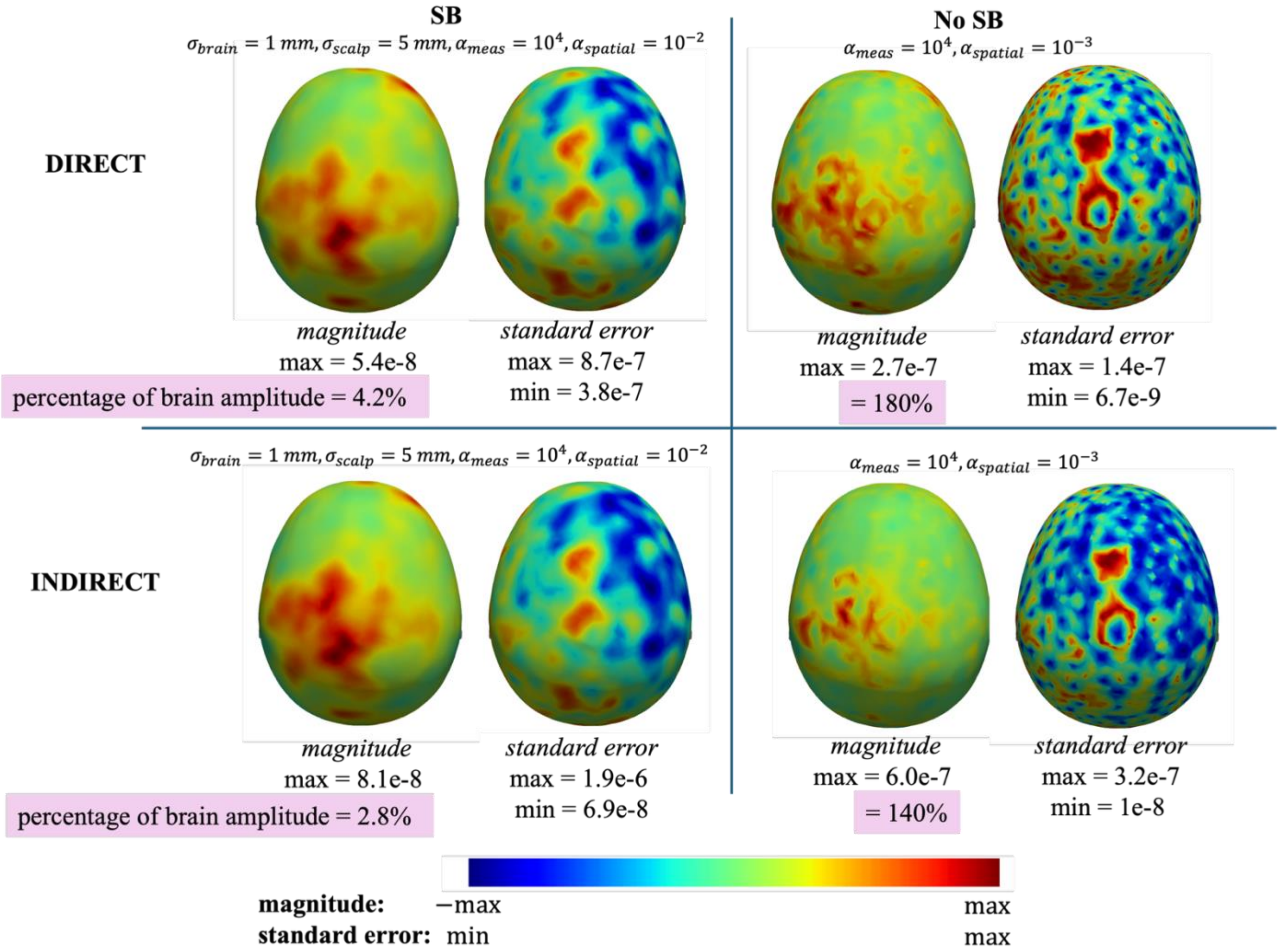

Supp Figure 4. Within each quadrant, the left image shows the magnitude of ΔHbO image on the scalp surface. The maximum amplitude is denoted below the image. The percentage of brain amplitude was calculated as the maximum of the scalp image divided by the maximum of the brain image. The right image shows the standard error of ΔHbO over the scalp surface. The maximum standard error is denoted below the image. ***Top Left:*** *Direct method, with spatial basis functions.* ***Top Right:*** *Direct method, no spatial basis functions.* ***Bottom Left:*** *Indirect method, with spatial basis functions.* ***Bottom Right:*** *Indirect method, no spatial basis functions. The effect of crosstalk between brain and scalp when not using spatial basis functions is evident since the maximum magnitude of the scalp is 180% and 140% greater than the brain for the direct and indirect methods respectively. Conversely, when spatial basis functions are used, the magnitude on the scalp is 4.2% and 2.8% for the direct and indirect methods respectively. The spatial frequency of the standard error is also different between the case when we have spatial basis functions and when we do not. The spatial basis functions on the scalp restrict the resolution of the noise we can reconstruct. This means only noise with a broad spatial distribution will be reconstructed on the scalp. When we do not use spatial basis functions, there is no prior on the spatial frequency of the noise we can reconstruct and as a result, higher frequency noise is reconstructed on the scalp.*
